# ﻿An overview of the genus *Lobothallia* (lichenized Ascomycota, Megasporaceae) in China

**DOI:** 10.3897/mycokeys.125.173554

**Published:** 2025-11-20

**Authors:** Lun Wang, Yi-Shan Feng, Li-Song Wang, Xin-Yu Wang, Yan-Yun Zhang

**Affiliations:** 1 Key Laboratory of Biodiversity Conservation and Ecological Security in the Yangtze River Basin of Anhui Province, College of Life Sciences, Anhui Normal University, 241000 Wuhu, China; 2 State Key Laboratory of Phytochemistry and Natural Medicines, Kunming Institute of Botany, Chinese Academy of Sciences, 650201 Kunming, China; 3 Yunnan Key Laboratory for Fungal Diversity and Green Development, Kunming Institute of Botany, Chinese Academy of Sciences, 650201 Kunming, China

**Keywords:** Lichen-forming fungi, new species, Pertusariales, phylogeny, species delimitation

## Abstract

*Lobothallia* is a species-rich genus of crustose lichens that is mainly distributed in the Northern Hemisphere. In recent years, significant advancements have been made in the taxonomy and phylogeny of this genus, leading to the description of numerous new species. However, *Lobothallia* in China has never been systematically revised. In this study, approximately 500 specimens of *Lobothallia* collected from different provinces of China were examined. Based on morphological and multi-gene phylogenetic analyses, an overview of this genus in China was provided. Eight species, *L. benzilanensis*, *L. complanata*, *L. polypycnidiata*, *L. pseudopruinosa*, *L. pulchra*, *L. rubra*, *L. stipitata* and *L. wangii*, were described as new to science. The species, *Lobothallia brachyloba*, was reported in China for the first time. In addition, we generated DNA sequences of the species *Lobothallia hedinii* from topotype specimens and clarified its phylogenetic position. Detailed descriptions, illustrations of morphological characters of the above-mentioned species, and comparisons with closely related taxa are provided. A dichotomous key to 22 species of *Lobothallia* from China is presented.

## ﻿Introduction

According to the estimation of Hyde et al. (2024), fungi comprise 19 phyla, 83 classes, 1,220 families, 10,685 genera and approximately 140,000 species. Lichenization represents a major fungal lifestyle, with ca. 19,000 known lichenized species (lichens) worldwide (Lücking et al. 2017), including about 3,000 reported from China (Wei 2020). Over the past decades, while China has witnessed a significant increase in lichenological research output, studies focusing on the genus *Lobothallia* (Clauzade & Cl. Roux) Hafellner remain scarce (Kou et al. 2013; Paukov et al. 2019; Zhang et al. 2020, 2024a).

The taxonomic concept of *Lobothallia* was initially established as a subgenus within *Aspicilia* A. Massal. (Clauzade and Roux 1984), with subsequent elevation to generic rank in Aspiciliaceae (ined.) by Hafellner (1991). Schmitt et al. (2006) reconstructed the phylogenetic framework of Pertusariales using nuclear large subunit (nuLSU) and mitochondrial small subunit (mtSSU) ribosomal DNA sequences, proposing the transfer of *Aspicilia* and *Lobothallia* to Megasporaceae Lumbsch and regarding Aspiciliaceae as a synonym of Megasporaceae. Nordin et al. (2010) reconstructed the phylogeny of Megasporaceae by integrating nrLSU and mtSSU data and confirmed the monophyly of both Megasporaceae and *Lobothallia*. Subsequent researchers have continually revised Megasporaceae, introducing several new genera (Sohrabi et al. 2013b; Haji Moniri et al. 2017; Zakeri et al. 2017; Paukov et al. 2024; Wheeler et al. 2024). To date, eleven genera within this family are accepted (Wheeler et al. 2024), with *Lobothallia* exhibiting the closest phylogenetic affinity to *Teuvoa* Sohrabi & S.D. Leav.

*Lobothallia* is widely distributed in arid and mountainous regions of the Northern Hemisphere, though species richness varies considerably across different parts of Eurasia (Paukov et al. 2019). A few widespread taxa, such as *Lobothallia alphoplaca* (Wahlenb.) Hafellner, have been recorded in the Southern Hemisphere, for example in New Zealand (Galloway and Ledingham 2012).

As the third most species-rich genus within Megasporaceae, *Lobothallia* currently encompasses 31 accepted species: *L. alphoplaca*, *L. brachyloba* Paukov & I.V. Frolov, *L. cernohorskyana* (Clauzade & Vězda) A. Nordin et al., *L. chadefaudiana* (Cl. Roux) A. Nordin et al., *L. cheresina* (Müll. Arg.) A. Nordin et al., *L. controversa* Cl. Roux & A. Nordin, *L. crassimarginata* Kou & Q. Ren, *L. crenulata* Lun Wang & Y.Y. Zhang, *L. densipruinosa* A. Ashraf et al., *L. determinata* (H. Magn.) T.B. Wheeler, *L. elobulata* Zulfiqar et al., *L. epiadelpha* Paukov & A. Nordin, *L. gangwondoana* S.Y. Kondr. et al., *L. hedinii* (H. Magn.) Paukov et al., *L. hydrocharis* (Poelt & Nimis) Sohrabi & Nimis, *L. iqbalii* Zulfiqar et al., *L. kuminovae* (Sedeln.) Sedeln., *L. lacteola* (Oxner) Şenkard. et al., *L. lobulata* Lun Wang & Y.Y. Zhang, *L. melanaspis* (Ach.) Hafellner, *L. pakistanica* Razzaq et al., *L. peltastictoides* (Hasse) T.B. Wheeler, *L. platycarpa* (J. Steiner) Paukov, *L. praeradiosa* (Nyl.) Hafellner, *L. pruinosa* Kou & Q. Ren, *L. pulvinata* R. Zulfiqar et al., *L. radiosa* (Hoffm.) Hafellner, *L. recedens* (Taylor) A. Nordin et al., *L. semisterilis* (H. Magn.) Y.Y. Zhang, *L. subdiffracta* (H. Magn.) Paukov and *L. zogtii* Paukov & Davydov (Hafellner 1991; Nordin et al. 2010; Roux 2012; Kou et al. 2013; Nimis 2016; Roux et al. 2016; Sedelnikova and Sedelnikov 2018; Paukov et al. 2019; Kondratyuk et al. 2020; Sipman and Aptroot 2020; Zhang et al. 2020, 2024a; Ashraf et al. 2022; Zulfiqar et al. 2022, 2023; Nascimbene et al. 2023; Wheeler et al. 2024). The taxonomic nomenclature of several species within the genus has undergone revision. *Lobothallia farinosa* (Flörke) A. Nordin, Savić & Tibell, originally proposed by Nordin et al. (2010) as a new combination based on the invalid basionym *Aspicilia farinosa* sensu Nyl., was subsequently reclassified by Roux et al. (2016) as the novel species *Lobothallia controversa*. *Lobothallia parasitica* (B. de Lesd.) Mayrhofer et al. (Mayrhofer et al. 2005) is considered a synonym of *L. radiosa*, since its basionym, *Aspicilia parasitica* B. de Lesd., was treated as *Lobothallia radiosa* chemotype *parasitica* by Paukov et al. (2019) based on isotype specimen study. Kou et al. (2013) described *Lobothallia helanensis* Kou & Q. Ren from Nei Mongol, China; however, subsequent phylogenetic and morphological analyses by Paukov et al. (2019) demonstrated that *Lecanora subdiffracta* H. Magn. and *Lobothallia helanensis* are conspecific. Since the epithet “*subdiffracta*” has nomenclatural priority, *Lobothallia helanensis* was recombined as *L. subdiffracta* (H. Magn.) Paukov.

In China, *Lobothallia* predominantly occurs in the arid and mountainous environments of northern and northwestern regions, as documented in both historical and contemporary studies (Magnusson 1940, 1944; Mamut et al. 2010, 2012; Kou et al. 2013; Paukov et al. 2019; Zhang et al. 2020, 2024a). Prior to this study, 13 species were known from China, with 10 species (i.e., *L. crassimarginata*, *L. cheresina*, *L. crenulata*, *L. determinata*, *L. hedinii*, *L. lobulata*, *L. pruinosa*, *L. semisterilis*, *L. subdiffracta*, and *L. zogtii*) having their type localities within the country. During our investigation of lichen diversity in China, approximately 500 *Lobothallia* specimens were examined and over 450 sequences (ITS, nrLSU, mtSSU) were generated. Using an integrative approach combining morphology, anatomy, chemistry, and multi-locus phylogenetic reconstruction, we conducted comprehensive molecular phylogenetic analyses and species delimitation within the genus. This investigation resulted in the discovery of eight new species and one new record for China, while also clarifying the phylogenetic position of *Lobothallia hedinii* for the first time. Detailed morphological descriptions with microscopic photographs are provided for these ten species, accompanied by interspecific comparative discussions. A dichotomous key to all Chinese *Lobothallia* species is presented.

## ﻿Materials and methods

### ﻿Sample collection, morphology and chemical examination

Through fieldwork, loans, and herbarium visits, approximately 500 *Lobothallia* specimens were examined in this study. These specimens were collected from nine provinces in China, namely Gansu, Hebei, Nei Mongol, Ningxia, Qinghai, Sichuan, Xizang, Xinjiang and Yunnan, covering the main distribution areas of this genus in the country. Voucher specimens are deposited in the following herbaria: Botany Herbarium, College of Life Sciences, Anhui Normal University (AHUB), Lichen Herbarium, Kunming Institute of Botany, Chinese Academy of Sciences (KUN-L) and Herbarium, College of Life Sciences, Shandong Normal University (SDNU). High-resolution images of type specimens for some species were provided by the Swedish Museum of Natural History (S) and the Komarov Botanical Institute of RAS (LE) or obtained from the website of Global Plants (https://plants.jstor.org/).

Specimens were examined using standard microscopy techniques. External morphological characters were observed on air-dried material under a Nikon SMZ 745T (Minato City, Japan) stereomicroscope. Anatomical features were studied using a Zeiss Axio Scope A1 light microscope (Oberkochen, Germany). Cross-sections of apothecia and thalli were cut by hand with a razor blade and observed after mounting in water, 10% aqueous solution of potassium hydroxide (K), 10% water solution of nitric acid HNO3 (N), Lugol’s iodine solutions (I), or lactophenol cotton blue (LCB). Lichen substances were preliminarily examined by spot tests using the following reagents: K, C (sodium hypochlorite solution), KC (C after pretreatment with K), and P (1,4-p-phenylenediamine). Further identification was conducted by thin layer chromatography (TLC) using solvent system C (Orange et al. 2001; Elix 2014).

In *Lobothallia*, the subhymenium and hypothecium are typically poorly delimited, even in sections stained with LCB. To avoid confusion, we therefore adopt the term “subhymenial layers” for the area between the lower limit of the hymenium and the base of the apothecium (Ivanovich et al. 2025). The separate plectenchyma atop the hymenium is called epithecium, which is different from the pigmented layer known as epihymenium (Bungartz 2002). Ascospore measurements are presented as: (lowest recorded–) (x̄ - SD) – x̄ – (x̄ + SD) (–highest recorded), where x̄ is the arithmetic mean and SD is the standard deviation, followed by the number of measurements (n). The length-to-width ration of ascospores is indicated as “l/w”.

### ﻿DNA extraction, PCR amplification and sequencing

Genomic DNA was extracted from dried specimens using the DNA secure Plant Kit (Tiangen, Beijing, China). PCR amplification was performed with the primers ITS1F and ITS 4a (Gardes and Bruns 1993; Larena et al. 1999) for the ITS region; LR0R and LR5 (Vilgalys and Hester 1990; Rehner and Samuels 1994) for the LSU/28S rDNA region; and mrSSU1 and mrSSU3R (Zoller et al. 1999) for the mitochondrial small subunit (mtSSU) rRNA region. Polymerase chain reaction (PCR) was carried out in a 25 μL volume, containing 12.5 μL 2 × Taq MasterMix (Aidlab, Hong Kong), 1 μL of each primer, 9.5 μL ddH_2_O and 1 μL DNA. The thermal cycling conditions followed Zhang et al. (2024b). PCR products were sequenced by Sangon Biotech (Shanghai, China) using the amplification primers.

### ﻿Sequence alignment and phylogenetic analyses

The raw sequences were initially verified using the BLAST tool on the NCBI online service (https://blast.ncbi.nlm.nih.gov/Blast.cgi) to confirm the lichen affinity. Forward and reverse sequences were assembled in Geneious Prime 2024.1 (https://www.geneious.com) and manually edited. We used newly generated nrITS, nrLSU and mtSSU sequences and additional sequences obtained from GenBank (Table 1) to construct a phylogenetic tree. The newly generated sequences have been deposited in GenBank.

**Table 1.** Voucher information and GenBank accession numbers of sequences used in this study. Newly generated sequences are in bold. ‘na’ indicates there is no sequence available.

Taxa	Locality	Voucher	nrITS	nrLSU	mtSSU
*Aspicilia angelica*	Montana, USA	Wheeler 3535	PP965029	PP965095	PP971393
*Aspicilia boykinii*	Montana, USA	Wheeler 7263	PP975030	PP965098	PP971376
*Aspicilia cinerea*	Finnmark, Norway	Wheeler 6277	MW447391	PP965054	MW424811
*Aspicilia epiglypta*	Västergötland, Sweden	Nordin 6303 (UPS)	EU057907	HM060756	HM060718
*Aspicilia fumosa*	Montana, USA	Wheeler 6802	PP965000	PP965068	PP971354
*Aspicilia indissimilis*	Sweden	Nordin 5943 (UPS)	EU057909	HM060746	HM060708
*Aspicilia olivaceobrunnea*	Arizona, USA	Owe-Larsson 8771 (ASU), type	PP965013	PP965084	PP971361
*Aspicilia subradians*	Alaska, USA	Wheeler 5070	PP965009	PP965080	PP971357
*Aspilidea myrinii*	Jämtland, Sweden	Nordin 7447 (WIS-0079196)	MW899516	MW907112	MW907106
*Aspilidea myrinii*	Sweden	Nordin 6205 (UPS)	na	HM060754	HM060716
*Aspilidea subadunans*	Alaska, USA	Wheeler 4628	MW899518	MW907114	MW907108
*Aspilidea subadunans*	Alaska, USA	Wheeler 3942	MW899517	MW907113	MW907109
*Aspiciliella cupreoglauca*	Greece	Sipman & Raus 61847 (B)	KY618843	KY576954	KY576930
*Aspiciliella intermutans*	Armenia	Zakeri 40503 (GLM)	KY596011	KY576947	KY576923
*Aspiciliella intermutans*	Czech Republic	Palice 11394 (PRA)	MH248855	MH255580	MH248888
*Aspiciliella portosantana*	Portugal	Sipman 63019 (B), type	KY618852	KY576962	KY576939
*Antidea brucei*	California, USA	Owe-Larsson 9161 (ASU), isotype	PP965011	PP965082	PP971359
*Antidea brucei*	California, USA	Knudsen 15069 (ASU)	PP975035	PP978599	PP974318
*Atrostelia magnifica*	Republic of Tyva, Russia	Davydov 21959 (LE), isotype	PP832009	PP832002	PP842134
*Atrostelia magnifica*	Republic of Tyva, Russia	Davydov 21961 (hb. Davydov), paratype	PP832010	PP832003	PP842135
*Circinaria arida*	Montana, USA	Wheeler 5819	PP964998	PP978597	PP971346
*C. caesiocinerea*	Uppland, Sweden	Tibell 22612 (UPS)	EU057897	HM060731	HM060693
*C. esculenta*	Astrakhan, Russia	Owe-Larsson 9796 (UPS)	JQ797511	JQ797493	JQ797485
*C. gibbosa*	Uppland, Sweden	Nordin 5878 (UPS)	EU057908	HM060740	HM060702
*C. hoffmannii*	Montana, USA	Wheeler 3634	PP964982	PP965052	PP971331
*C. leprosescens*	Sweden	Nordin 5906 (UPS)	EU057911	HM060749	HM060711
*Lobothallia alphoplaca*	Ukraine	SK A20 (KW)	KT456207	na	KT456211
*L. alphoplaca*	Norway	O–L–200411	MK812484	na	na
*L. benzilanensis*	China, Yunnan	18-60350 (KUN-L)	**PV956002**	**PV955950**	na
*L. brachyloba*	Russia, Republic of Altai	Frolov 357 (UFU), holotype	MK347506	na	MK348228
*L. brachyloba*	China, Xinjiang	22-72815 (KUN-L)	**PV956003**	na	na
*L. cernohorskyana*	Iran, South Khorasan	Tari 2311 (B)	na	JQ797496	JQ797481
*L. cheresina*	USA, Utah	Wheeler 7216 (hb. Wheeler)	PP965022	na	PP971366
*L. complanata*	China, Sichuan	20-67554 (KUN-L), holotype	**PV956004**	**PV955951**	**PV956066**
*L. complanata*	China, Sichuan	20-66561 (KUN-L)	**PV956005**	na	**PV956067**
*L. controversa*	France, Rhône-Alpes	Roux 25286 (UPS), holotype	na	HM060761	HM060723
*L. crassimarginata*	China, Nei Mongol	Wang 20122565 (SDNU), holotype	JX476026	na	na
*L. crassimarginata*	China, Nei Mongol	Tong 20122583 (SDNU)	KC007439	na	na
*L. crenulata*	China, Xizang	ZYY22-301 (KUN-L), holotype	PP663142	**PV955952**	PP663165
*L. crenulata*	China, Xizang	ZYY22-331 (AHUB)	PP663141	**PV955953**	PP663164
*L. densipruinosa*	Pakistan	LAH 36790, holotype	MZ871507	na	na
*L. densipruinosa*	Pakistan	LAH 36951	MZ871515	na	na
*L. determinata*	USA, Montana	Wheeler 3641 (hb. Wheeler)	PP965020	na	PP971365
*L. determinata*	USA, Montana	Wheeler 6017 (hb. Wheeler)	PP965021	na	PP971398
*L. elobulata*	Pakistan	LAH37153, holotype	ON384441	na	na
*L. elobulata*	Pakistan	LAH37154	ON428667	na	na
*L. epiadelpha*	Russia, Orenburg Oblast	Paukov 1881 (UFU L-3189), holotype	MK347505	na	MK348232
*L. hedinii*	China, Gansu	18-59518 (KUN-L)	**PV956006**	na	na
*L. hedinii*	China, Qinghai	20-68298 (KUN-L)	**PV956008**	na	**PV956069**
*L. hedinii*	China, Xizang	22-71155 (KUN-L)	**PV956016**	**PV955960**	**PV956077**
*L. hedinii*	China, Qinghai	20-68147 (KUN-L)	**PV956019**	**PV955962**	**PV956080**
*L. hydrocharis*	Italy, Sardinia	JN72085b (BOLO)	OQ073922	na	na
*L. hydrocharis*	Italy, Sardinia	SMNS-STU-F-0002807 (STU)	OQ073923	na	na
*L. iqbalii*	Pakistan	LAH37149, holotype	ON384444	na	na
*L. iqbalii*	Pakistan	LAH37150	ON384445	na	na
*L. lobulata*	China, Sichuan	ZYY22-819 (KUN-L), holotype	PP663143	**PV955963**	PP663166
*L. lobulata*	China, Sichuan	ZYY22-829 (AHUB)	PP663147	**PV955964**	PP663170
*L. melanaspis*	Sweden, Jämtland	Nordin 6622 (UPS)	HQ259272	HM060726	HM060688
*L. melanaspis*	Norway	Owe-Larsson 8943a (UPS)	JF825524	na	na
*L. pakistanica*	Pakistan	LAH37137, holotype	ON392718	na	na
*L. pakistanica*	Pakistan	LAH37139	ON392720	na	na
*L. peltastictoides*	USA, California	Knudsen 14420 (UCR)	PP965019	PP965087	PP971367
*L. polypycnidiata*	China, Qinghai	XY20-3034 (KUN-L), holotype	**PV956020**	**PV955965**	**PV956081**
*L. polypycnidiata*	China, Qinghai	XY20-808 (KUN-L)	**PV956021**	**PV955966**	**PV956082**
*L. polypycnidiata*	China, Qinghai	20-68413 (KUN-L)	**PV956022**	na	**PV956083**
*L. polypycnidiata*	China, Qinghai	20-67303 (KUN-L)	**PV956023**	na	**PV956084**
*L. polypycnidiata*	China, Qinghai	20-67134 (KUN-L)	**PV956024**	**PV955967**	**PV956085**
*L. polypycnidiata*	China, Qinghai	20-68672 (KUN-L)	**PV956025**	na	**PV956086**
*L. polypycnidiata*	China, Qinghai	20-67160 (KUN-L)	**PV956026**	**PV955968**	**PV956087**
*L. polypycnidiata*	China, Xizang	20-68878 (KUN-L)	**PV956027**	**PV955969**	na
*L. praeradiosa*	China, Xinjiang	ZYY22-570 (AHUB)	PP663151	**PV955970**	PP663173
*L. praeradiosa*	China, Xinjiang	ZYY22-596 (AHUB)	PP663150	**PV955971**	PP663172
*L. praeradiosa*	Russia, Orenburg oblast	Paukov (UFU L-1264)	MK347501	na	MK348229
*L. pruinosa*	China, Qinghai	22-73170 (KUN-L)	**PV956030**	**PV955972**	**PV956090**
*L. pruinosa*	China, Nei Mongol	Wang 20123278 (SDNU), holotype	JX476028	na	na
*L. pseudopruinosa*	China, Xizang	19-64075 (KUN-L), holotype	**PV956031**	**PV955973**	na
*L. pseudopruinosa*	China, Qinghai	20-68331 (KUN-L)	**PV956032**	**PV955974**	na
*L. pseudopruinosa*	China, Qinghai	XY22-1089 (KUN-L)	**PV956033**	**PV955975**	na
*L. pseudopruinosa*	China, Qinghai	22-72438 (KUN-L)	**PV956034**	**PV955976**	**PV956091**
*L. pseudopruinosa*	China, Xizang	ZYY22-324 (KUN-L)	**PV956035**	**PV955977**	na
*L. pseudopruinosa*	China, Qinghai	20-67831 (KUN-L)	**PV956036**	**PV955978**	**PV956092**
*L. pulchra*	China, Xizang	19-66027 (KUN-L), holotype	**PV956037**	na	**PV956093**
*L. pulchra*	China, Qinghai	XY20-3169 (KUN-L)	**PV956038**	na	**PV956094**
*L. pulvinata*	Pakistan	CH-93, paratype	OP268463	na	na
*L. pulvinata*	Pakistan	PN-04	OP268468	na	OQ772296
*L. radiosa*	Sweden	Nordin 5889 (UPS)	JF703124	na	na
*L. radiosa*	Czech Republic, Southern Moravia	Kocourkova 10226/1	ON447612	ON391443	ON367885
*L. radiosa*	Switzerland	Lumbsch 9 Aug. 2004 (F)	na	DQ780306	DQ780274
*L. radiosa*	China, Xinjiang	22-72256 (KUN-L)	**PX061735**	na	na
*L. recedens*	Portugal	Sipman 62857	MN586980	na	na
*L. recedens*	Sweden	Nordin 6035 (UPS)	HQ406807	na	na
*L. rubra*	China, Xinjiang	22-72301 (KUN-L)	**PV956039**	**PV955979**	**PV956095**
*L. rubra*	China, Xinjiang	XY22-897 (KUN-L), holotype	**PV956040**	na	**PV956096**
*L. rubra*	China, Xinjiang	22-72886 (KUN-L)	**PV956041**	na	**PV956097**
*L. semisterilis*	China, Gansu	ZYY22-704 (AHUB)	PP663161	**PV955980**	PP663180
*L. semisterilis*	China, Gansu	ZYY22-715 (AHUB)	PP663159	**PV955981**	PP663178
*L. subdiffracta*	Russia, Republic of Altai	Frolov 178-1 (UFU)	MK347503	na	MK348233
*L. subdiffracta*	China, Xinjiang	ZYY22-628 (AHUB)	PP663162	na	PP663181
‘*L. helanensis*’	China, Nei Mongol	D.B. Tong 20122518 (SDNU)	JX476030	na	na
‘*L. helanensis*’	China, Nei Mongol	D.B. Tong 20122791 (SDNU)	JX476031	na	na
L. subdiffracta var. rimosa	China, Xinjiang	ZYY22-647 (KUN-L)	PP663156	**PV955982**	PP663175
L. subdiffracta var. rimosa	China, Xinjiang	Wang et al. 22-72975 (KUN-L)	PP663155	na	PP663174
*L. stipitata*	China, Xinjiang	22-72871 (KUN-L), holotype	**PV956044**	na	**PV956100**
*L. stipitata*	China, Xinjiang	ZYY22-616 (KUN-L)	**PV956045**	**PV955983**	**PV956101**
*L. stipitata*	China, Xinjiang	ZYY22-617 (KUN-L)	**PV956046**	**PV955984**	**PV956102**
*L. stipitata*	China, Xinjiang	XY22-887 (KUN-L)	**PV956047**	**PV955985**	**PV956103**
*L. wangii*	China, Xizang	22-71215 (KUN-L), holotype	**PV956048**	na	na
*L. wangii*	China, Xizang	ZYY22-334 (KUN-L)	**PV956049**	**PV955986**	**PV956104**
*L. wangii*	China, Xizang	19-65657 (KUN-L)	**PV956050**	na	**PV956105**
*Megaspora rimisorediata*	Alberta, Canada	Spribille WP642	PP964997	PP978600	na
*M. verrucosa*	Antarctica	Zuo NJ2018074 (hb. unknown)	OL331496	OL331779	na
*M. verrucosa*	East Azerbaijan, Iran	Sipman 55434 (B)	na	JQ797498	JQ797483
*Ochrolechia subpallescens*	USA	Lumbsch 19900a (MIN)	na	GU980985	GU980978
*Ochrolechia upsaliensis*	USA	Lumbsch 19916e (MIN)	na	GU980986	GU980979
*Ochrolechia yasudae*	na	AFTOL 882	na	DQ986776	DQ986902
*Oxneriaria haeyrenii*	Sweden	Nordin 5997 (UPS)	na	HM060755	HM060717
*Oxneriaria mashiginensis*	Sweden	Nordin 5790 (UPS)	EU057912	HM060732	HM060694
*Oxneriaria permutata*	Sweden	Nordin 6027 (UPS)	EU057918	HM060747	HM060709
*Oxneriaria supertegens*	Sweden	Nordin 6023 (UPS)	EU057938	HM060751	HM060713
*Sagedia mastrucata*	Finnmark, Norway	Wheeler 6467	PP975034	PP965066	PP971347
*S. simoensis*	Troms, Norway	Owe-Larsson 9000 (UPS)	EU057926	HM060739	HM060701
*S. simoensis*	Finnmark, Norway	Wheeler 6288	PP975033	PP965067	PP971348
*S. zonata*	Troms, Norway	Owe-Larsson 8942 (UPS)	EU057946	HM060738	HM060700
*Teuvoa junipericola*	Utah, USA	Wheeler 7390	PP965018	na	PP971364
*T. junipericola*	Utah, USA	Leavitt 742 (BRY)	JX306744	JX306758	na
*T. uxoris*	Castilla, Spain	Rico & Pizarro 3622 (H)	JX306743	JX306757	na
*T. uxoris*	Castilla, Spain	Rico & Pizarro 765 (BRY)	JX306745	JX306759	na

Matrixes of nrITS, nrLSU, mtSSU were aligned separately using MAFFT v. 7 (https://mafft.cbrc.jp/alignment/server/) with the E-INS-I strategy (Katoh and Standley 2013; Katoh et al. 2019) under default parameters. The alignments were checked in MEGA v. 7 (Kumar et al. 2016) and minor misalignments were manually adjusted. Subsequently, Maximum Likelihood (ML) single-locus trees were constructed in IQTree (Nguyen et al. 2015; Trifinopoulos et al. 2016) and compared to assess potential, strongly supported conflicts among gene trees. When all statistically supported clades (bootstrap support values≥70%) were consistent across individual gene trees, the datasets were concatenated. The three loci were combined via the Concatenate Sequence function in PhyloSuite v1.2.3 (Xiang et al. 2023). ModelFinder (Kalyaanamoorthy et al. 2017) was used to estimate the best schemes and nucleotide substitution models for maximum likelihood (ML) and Bayesian inference (BI) analyses. The best schemes and selected models are shown in Table 2.

**Table 2.** The best schemes and nucleotide substitution models selected by ModelFinder for the concatenated 3-gene dataset.

Partition	Regions	Positions	Model for IQ-TREE	Model for Bayes
Partition 1	ITS1	1–238	TIM2e+I+G4	GTR+F+I+G4
Partition 2	5.8S	239–395	TNe+I+G4	K2P+I
Partition 3	ITS2	396–575	TIM2e+I+G4	GTR+F+I+G4
Partition 4	nrLSU	576–1669	GTR+F+I+G4	GTR+F+I+G4
Partition 5	mtSSU	1670–2342	GTR+F+I+G4	GTR+F+I+G4

Bayesian tree inference was carried out by MrBayes 3.2 (Ronquist et al. 2012). Two independent runs of four Markov Chain Monte Carlo (MCMC) were performed each for 5 M generations, sampling every 1000 generation. To assess convergence, the average standard deviation of split frequencies (ASDSF) among runs was calculated every 200 generations, with the first 25% of sampled trees discarded as burn-in. The analyses were terminated automatically when the ASDSF values fell below 0.01, indicating that stationarity had been reached. ML tree analyses were performed using IQ-TREE (Nguyen et al. 2015; Trifinopoulos et al. 2016) under default settings, with branch support evaluated through 1000 standard non-parametric bootstrap replicates. Trees were visualized and graphically improved using Interactive Tree of Life webpage (iTOL) (Letunic and Bork 2021) and Adobe illustrator 2020 SP.

## ﻿Results

### ﻿Phylogenetic analysis

**Figure 1.**
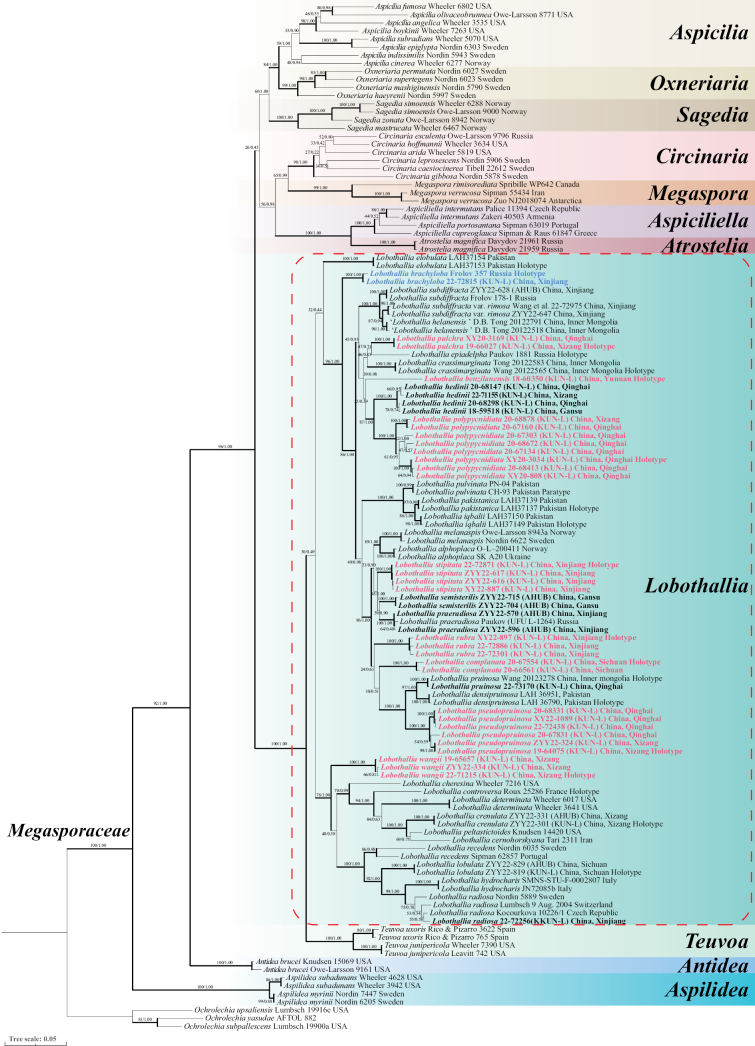
Bayesian phylogenetic tree of *Lobothallia* inferred from the concatenated alignment of nrITS-nrLSU-mtSSU sequences. Maximum likelihood bootstrap values (MLBP) and Bayesian posterior probabilities (BPP) are indicated at the nodes. Thickened branches indicate MLBP ≥ 70% and BPP ≥ 0.95. Newly described species are highlighted in red, newly recorded species in China in blue, and newly generated sequences in bold.

A phylogenetic tree of *Lobothallia* was reconstructed based on a concatenated matrix of nrITS, nrLSU, and mtSSU sequences. *Ochrolechia subpallescens* Verseghy, *O. upsaliensis* (L.) A. Massal., and *O. yasudae* Vain. from the family Ochrolechiaceae R.C. Harris ex Lumbsch & I. Schmitt were selected as outgroups (Wheeler et al. 2024). The dataset comprised 127 samples, 83 of which belonged to the genus *Lobothallia*, including 20 type specimens and 45 newly sequenced samples (Table 1). The concatenated alignment contained 118 nrITS (36 newly generated), 78 nrLSU (30 new), and 91 mtSSU (26 new) sequences, with a total length of 2342 bp partitioned into five regions (Table 2). Phylogenetic reconstruction was performed under Maximum Likelihood (ML) and Bayesian Inference (BI) methods. Both methods produced congruent tree topologies; the BI tree is presented in the main text (Fig. 1). Statistical support values are labeled on the branch when the ML bootstrap proportion (MLBP) ≥ 70% and Bayesian posterior probabilities (BPP) ≥ 0.95.

The phylogenetic analysis resolved 33 species of *Lobothallia* into three major clades (Clade I–III). Clade I (MLBP/BPP ≥96/1.00) represents the core group of *Lobothallia*, exhibiting phenotypic diversity spanning the genus. The basal lineage of this clade is *Lobothallia brachyloba*, characterized by its short marginal lobes, 1–7 apothecia per areole, and cryptolecanorine apothecia (Paukov et al. 2019). Most species within Clade I formed highly supported monophyletic groups, with the exception of *L. epiadelpha* and *L. benzilanensis*, which remained as singletons. Some backbone nodes, however, received only weak support, potentially indicating the insufficient informative sites of the sequences. Within Clade I, eight novel lineages were identified: seven described as new species, one confirmed as the species *L. hedinii* that was originally described from Gansu Province, China, for which no sequence was available before this research. *Lobothallia pulchra* and *L. crassimarginata* clustered together with weak support; both species contain stictic acid but differ in thallus color and apothecial type. Another new species, *Lobothallia stipitata*, also contains stictic acid but is distinguished by its pale brown to brown thallus, and its distinct pruinose apothecial disc with a brown thalline margin. *Lobothallia polypycnidiata* and *L. hedinii* formed sister lineages with strong support (87/1.00). Although several short-branched, highly supported subclades were observed within *L. polypycnidiata*, we treated them as the same species due to their consistent morphology and overlapping geographical distributions, following the species delimitation framework of Carstens et al. (2013). *Lobothallia rubra* and *L. complanata* clustered together with weak support but are morphologically distinct, differing in thallus color and thickness (white and thick vs. orangish gray and thin) and the presence versus absence of gyrophoric acid. *Lobothallia pseudopruinosa*, *L. pruinosa*, and *L. densipruinosa* gathered in a subclade with high support (100/1.00).

Clade II consisted solely of *Lobothallia elobulata*. This clade is distinguished from Clades I and III by the presence of prothallus, a characteristic commonly found in the genus *Aspicilia* within Megasporaceae. Clade III (76/1.00) was resolved as a monophyletic lineage with *Lobothallia wangii* at the base. Within this clade, *Lobothallia hydrocharis*, *L. lobulata*, *L. radiosa*, and *L. recedens* formed a strongly supported subclade (100/1.00), in which all species consistently displayed gray to dark gray thalli. The remaining species, *Lobothallia cernohorskyana*, *L. cheresina*, *L. crenulata*, *L. determinata*, and *L. peltastictoides*, formed a distinct, well-supported subclade (70/0.99) characterized by white to off-white thalli.

### ﻿Taxonomy

#### ﻿New species

##### ﻿ Lobothallia benzilanensis

Taxon classificationFungiPertusarialesMegasporaceae

Lun Wang & Y. Y. Zhang sp. nov.

42A3EBD0-D85C-53D5-9153-B82F090BDCD1

Fungal Names: FN 572942

Fig. 2

###### Etymology.

The specific epithet refers to Benzilan Town, the type locality of this species.

###### Diagnosis.

Thallus non-lobate, areoles dispersed to continuous, tightly adnate to the substrate; upper surface pruinose, off-white, partially with orangish tinge; apothecia cryptolecanorine to lecanorine; containing norstictic, cryptostictic, and connorstictic acids.

###### Holotype.

China • Yunnan Prov.: Deqin Co., Benzilan Town, 28°11'35.79"N, 99°21'07.65"E, Along Jinshajiang River side, alt. 2108 m, on rock, 19 August 2018, Li-Song Wang et al. 18-60350 (KUN-L 63843).

###### Description.

Thallus areolate, non-lobate, tightly adnate to the substrate. Areoles dispersed to continuous, central areoles irregular, 0.5–1 mm wide, marginal areoles usually larger than the center, 3–10 mm wide, cracked; distant simple areoles circular, 0.5–3 mm wide. Upper surface plane to slightly convex, pruinose, off-white, with orangish tinge. Upper cortex paraplectenchymatous, 30–60 µm thick, inspersed with brown to dark brown granules (soluble in K); epinecral layer gelatinous, 10–20 µm thick, with hyaline crystals and dark brown granules (insoluble in K). Algal layer 50–125 µm thick, discontinuous; photobiont chlorococcoid, cells 8–20 µm diameter. Medulla opaque, filled with gray-black granules (not or partially soluble in K). Lower cortex absent.

Apothecia common (1–4 per areole), orbicular, 0.3–1 mm diameter, initially cryptolecanorine, becoming lecanorine and elevated above areoles at maturity, not constricted at base; disc slightly concave to plane, matt, black, pruinose; apothecial margin indistinct when young, becoming prominent (0.05–0.15 mm wide) during development, receding with disc expansion. Exciple absent or narrow, not exceeding 50 μm. Epithecium, hymenium and subhymenial layers combined 175–200 µm high; epithecium 10–20 µm high, containing hyaline crystals; epihymenium with brown granules (soluble in K) and black granules (insoluble in K), N−; hymenium 100–120 µm high, hyaline, I+ blue; subhymenial layers 50–75 µm high, hyaline, I+ blue; algal layer sparse below hypothecium; paraphyses simple, septate, non-moniliform, with 1–3 uppermost cells wider than the basal cells, 4–6 μm wide (basal cells ca. 2 μm wide); asci clavate, *Aspicilia*-type, 8-spored, 70–80 × 18–25 µm; ascospores simple, hyaline, broadly ellipsoid, (10.0-)11.5–12.4–13.2(-14.0) × (8.0-)7.9–8.6–9.3(-10.0) µm (n = 36), l/w ratio (1.2-)1.3–1.4–1.5(-1.7), wall ca.1 µm thick. Pycnidia few, punctiform, plane to slightly convex, 0.05–0.2 mm diameter; ostiole brown to dark brown; conidia bacilliform, hyaline, 4–6 × 1 µm.

**Figure 2.**
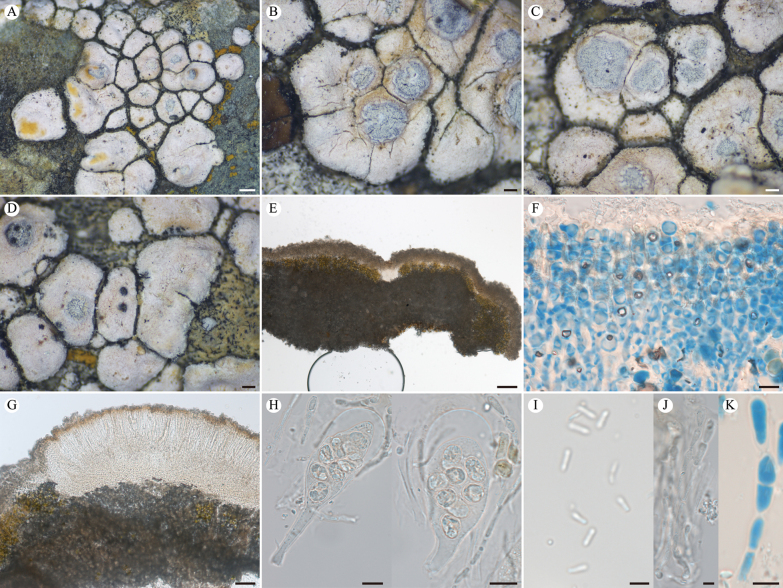
*Lobothallia benzilanensis* (KUN-L 63843, holotype). **A.** Thallus; **B, C.** Apothecia; **D.** Punctiform pycnidia; **E.** Section of thallus; **F.** Upper cortex and epinecral layer (LCB); **G.** Vertical section of apothecium; **H.** Asci and ascospores; **I.** Conidia; **J, K.** Paraphyses (LCB after K). Scale bars: 0.5 mm (**A**); 0.2 mm (**B, C, D**); 100 µm (**E**); 50 µm (**G**); 10 µm (**F, H**); 5 µm (**I**, **J**, **K**).

###### Chemistry.

Cortex K+ yellow, P−; Medulla K+ yellow to orangish red, P+ yellow, C−, KC−; norstictic, cryptostictic and connorstictic acids detected in TLC.

###### Habitat and distribution.

Growing on the exposed calcareous rock. Currently only known from the town of Benzilan, Yunnan, China.

###### Notes.

This species is similar to *Lobothallia gangwondoana* in having a non-lobate thallus with dispersed areoles. However, *L. gangwondoana* differs in its larger areoles, epruinose and slightly brownish-gray to cacao-gray upper surface (vs. pruinose, off-white with light orangish tinge in *L. benzilanensis*), epruinose apothecial disc (vs. pruinose), and in the lack of cryptostictic and connorstictic acids (Kondratyuk et al. 2020).

###### Additional specimens examined.

China • Yunnan Prov.: Diqing Tibetan Autonomous Prefecture, Shangri-La City, near Jinshajiang River in Benzilan Vil., 28°11'35.79"N, 99°21'07.65"E, alt. 2108 m, on rock, 19 August 2018, Li-Song Wang et al. 18-60354 (KUN-L 63847).

##### ﻿ Lobothallia complanata

Taxon classificationFungiPertusarialesMegasporaceae

Lun Wang & Y. Y. Zhang sp. nov.

1BC24AD2-51C8-58E1-8A12-B01E6210AC6C

Fungal Names: FN 572943

Fig. 3

###### Etymology.

The epithet refers to the flat thallus of this species.

###### Diagnosis.

Thallus appressed to the substrate, areolate with a flat and radiately lobate margin; upper surface orangish gray, covered by white pruina; apothecia lecanorine, orbicular, adnate, discs brown with orangish pruina; mature pycnidia protruding.

###### Holotype.

China • Sichuan Prov.: Aba Tibetan and Qiang Autonomous Prefecture, Li Co., Putou Vil., 31°25'08.23"N, 103°04'10.85"E, alt. 2099 m, on rock, 4 September 2020, Li-Song Wang et al. 20-67554 (KUN-L 75731).

###### Description.

Thallus areolate with lobate margin, up to 5 cm across, closely appressed to the substrate, 0.3–1.5 mm thick. Areoles polygonal to suborbicular, (0.3–)0.5–1.2(–2.0) mm wide, interspaces between areoles 0.05–0.1 mm wide. Lobes flat, ca. 0.3 mm thick, elongate, 2–3(–3.5) mm long, 0.4–0.8(–1.0) mm wide at base, 0.6–1.5 mm wide at apex. Upper surface matt, orangish gray, covered by white pruina. Upper cortex paraplectenchymatous, even, 20–30 μm thick, with brown granules (soluble in K); epinecral layer 10–15 µm thick, with dense black granules (insoluble in K). Algal layer 75–125 µm thick, discontinuous; photobiont chlorococcoid, cells 8–20 µm diameter. Medulla opaque, filled with gray-black granules. Lower cortex absent.

Apothecia lecanorine, scattered, orbicular, (0.4–)0.8–2.0(–2.5) mm in diameter, adnate, constricted at base at maturity; disc plane to slightly convex, matt, brown with orangish pruina; apothecial margin entire, orange-gray, pruinose, persistent, 0.1–0.25 mm wide. Exciple narrow, widening to 20–60 μm in the uppermost part. Epithecium, hymenium and subhymenial layers combined 130–180 µm high; epithecium 10–15 µm high; epihymenium 10–20 μm high, with brown to dark brown granules (mostly soluble in K, leaving faint brown residue), N−; hymenium 75–100 µm high, hyaline, I+ blue; subhymenial layers 40–75 μm high, hyaline, I+ blue; algal layer discontinuous below hypothecium, 20–50 μm high; paraphyses simple, rarely anastomosed, septate, submoniliform, with 2–3 uppermost cells shorter and wider than the basal cells, 3–4 μm wide (basal cells ca. 2 μm wide); asci clavate, *Aspicilia*-type, hyaline, 8-spored, 60–70 × 20–25 µm; ascospores simple, hyaline, broadly ellipsoid to spherical, (9.0-)9.5–10.4–11.3(-12.0) × (7.0-)7.7–8.7–9.8(-10.0) µm (n = 49), l/w ratio (1.0-)1.0–1.2–1.4(-1.6), wall ca.1 µm thick. Pycnidia common, 0.2–0.5 mm diameter, distinctly protruding at maturity; ostiole brownish to dark brown; conidia bacilliform, hyaline, 4–6 × 1 µm.

**Figure 3.**
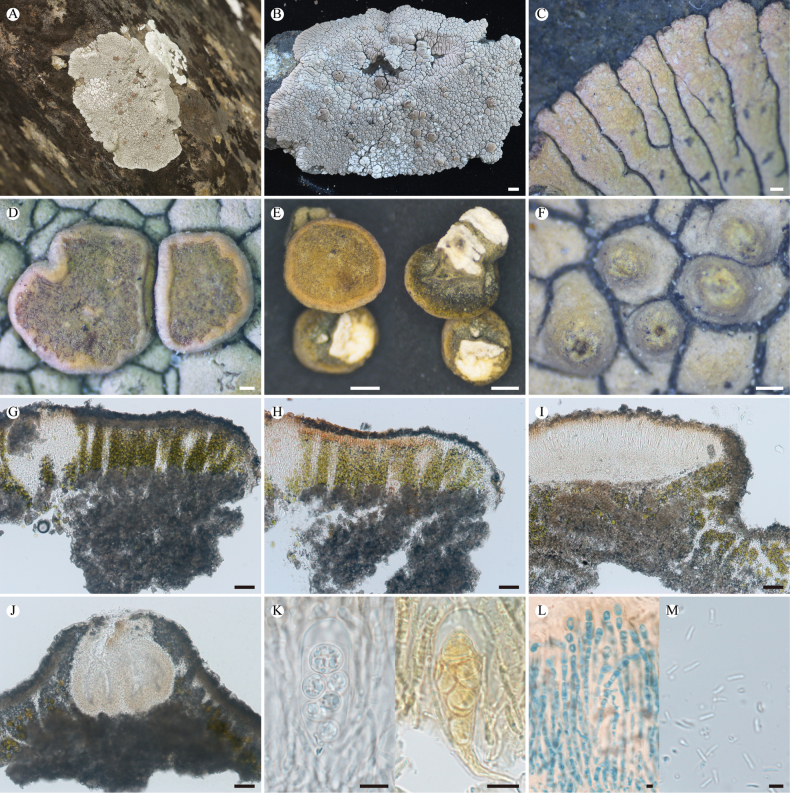
*Lobothallia complanata* (KUN-L 75731, holotype). **A.** Habitat; **B.** Thallus; **C.** Lobes; **D, E.** Apothecia; **F.** Protruding pycnidia; **G, H.** Section of thallus (H in K reagent); **I.** Vertical section of apothecium; **J.** Vertical section of pycnidium; **K.** Asci and ascospores (right in Lugol’s solution); **L.** Paraphyses (LCB); **M.** Conidia. Scale bars: 2 mm (**B**); 0.5 mm (**E**); 0.2 mm (**C, D, F**); 50 µm (**G, H, I, J**); 10 µm (**K**); 5 µm (**L, M**).

###### Chemistry.

Cortex K+ yellow to red, P−; Medulla K+ yellow to red, P+ yellow, C−, KC−; norstictic and connorstictic acids detected by TLC.

###### Habitat and distribution.

Saxicolous. Currently only known in Sichuan Prov., China.

###### Notes.

*Lobothallia lobulata* resembles the new species in the flat and appressed thallus. However, *L. lobulata* is readily distinguished by its light gray thallus surface bearing lobules, epruinose apothecial disc and the absence of secondary metabolites (Zhang et al. 2024a).

###### Additional specimens examined.

China • Sichuan Prov.: Aba Tibetan and Qiang Autonomous Prefecture, Li Co., Putou Vil., 31°25'08.23"N, 103°04'10.85"E, alt. 2099 m, on rock, 4 September 2020, Li-Song Wang et al. 20-66561 (KUN-L 74736) • Rangtang Co., 32°17'39.37"N, 101°00'44.83"E, alt. 3535 m, on rock, 4 September 2020, Xin-Yu Wang et al. XY20-2551 (KUN-L 78906).

##### ﻿ Lobothallia polypycnidiata

Taxon classificationFungiPertusarialesMegasporaceae

Lun Wang & Y. Y. Zhang sp. nov.

22F84E4B-75A5-54B8-A396-A8C8B1083C92

Fungal Names: FN 572944

Fig. 4

###### Etymology.

The epithet refers to the numerous pycnidia per areole of this species.

###### Diagnosis.

Thallus areolate, with radiate marginal lobes, pycnidia prominent, usually numerous pycnidia per areole, curved and elongate. This species colonizes diverse substrates, predominantly calcareous and siliceous rocks, with an occasional occurrence on a coniferous stump.

###### Holotype.

China • Qinghai Prov.: Qumalai Co., Qumahe Vil., 34°54'53.01"N, 94°46'17.29"E, alt. 4412 m, on rock, 17 September 2020, Xin-Yu Wang et al. XY20-3034 (KUN-L 79390).

###### Description.

Thallus adnate to the substratum, up to 5–7 cm across, centrally areolate, 2–4 mm thick, marginally lobate, 0.5–1 mm thick. Areoles (0.5–)0.7–1(–1.5) mm wide, angular to rounded, slightly constricted at base, interspaces between areoles (0.05–)0.1–0.2(–0.4) mm wide, surface flat to slightly convex, grayish-brown covered with white pruina. Lobes simple to irregularly branched, radiate, not overlapping, (1–)2–4(–5) mm long, (0.6–)1–1.5 mm wide, surface pale brown with thin pruina. Upper cortex paraplectenchymatous, 30–50 μm thick, with brown granules (soluble in K); epinecral layer 10–20 µm thick, gelatinous with dark granules (insoluble in K). Algal layer (75–)100–150(–250) μm thick, discontinuous; photobiont chlorococcoid, cells 8–16 μm diam. Medulla 0.5–1 mm thick, opaque, filled with gray-black granules (insoluble or partially soluble in K). Lower cortex absent.

Apothecia lecanorine, numerous, 1–3 per areole, (0.3–)0.8–2(–2.5) mm in diameter, clustered or scattered, orbicular to angular by pressure, not to slightly constricted at base; disc plane to slightly concave, black or brown, epruinose (rarely with thin pruina); apothecial margin entire, concolorous with thallus, slightly elevated, persistent but narrowing with disc expansion, 0.15–0.25 mm wide. Exciple narrow, 40–50 μm in the uppermost part. Epithecium, hymenium and subhymenial layers combined 125–150 µm high; epithecium 10–15 µm high; epihymenium 15–25 μm high, with brown (soluble in K) and pale brown (insoluble in K) granules, N−; hymenium 70–100 µm high, hyaline, I+ blue; subhymenial layers 40–80 μm high, hyaline, I+ blue, hypothecium 30–40 μm, algal layer discontinuous below hypothecium; paraphyses simple, septate, submoniliform to moniliform at the tips, with 2–5 uppermost cells shorter and wider than the basal cells, 4–6 μm wide (basal cells ca. 2 μm wide); asci clavate, *Aspicilia*-type, hyaline, 8-spored, 60–70 × 20–30 µm; ascospores simple, hyaline, broadly ellipsoid to ellipsoid, (10.0-)11.2–12.0–12.8(-13.0) × (7.0-)7.0–7.8–8.5(-9.0) µm (n = 38), l/w ratio (1.1-)1.4–1.6–1.8(-1.9), wall ca.1 µm thick. Pycnidia numerous, usually numerous pycnidia per areole, immersed, unilocular to multilocular, spot-like, 0.1–0.3 mm when young, shortly elongate to curved, 0.4–0.5 × 0.2–0.3 mm at maturity; ostioles dark brown to blackish; conidia bacilliform, hyaline, 5–6(–7) × 1 µm.

**Figure 4.**
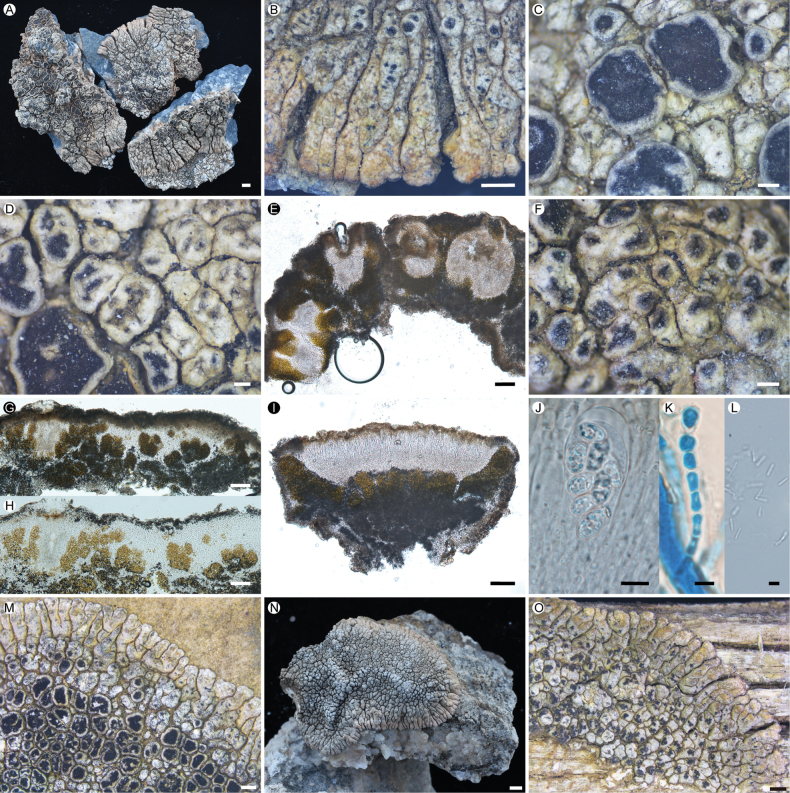
*Lobothallia polypycnidiata*. **A–L.** XY20-3034 (KUN-L 79390, holotype): **A.** Thallus; **B.** Lobes; **C.** Apothecia; **D.** Curved pycnidia; **E.** Vertical section of thallus and pycnidia; **F.** Densely growing pycnidia; **G, H.** Section of thallus (H in K reagent); **I.** Vertical section of apothecium; **J.** Ascus and ascospores; **K.** Paraphyses (LCB); **L.** Conidia. **M–N.** Other specimens: **M.** 20-68583, on calciferous rock; **N.** 20-68878, on calciferous rock; **O.** 20-68672, on organic substrate. Scale bars: 2 mm (**A, N**); 1 mm (**B, M, O**); 0.25 mm (**C, D, F**); 100 µm (**E, G, H, I**); 10 µm (**J**); 5 µm (**K, L**).

###### Chemistry.

Cortex K+ yellow to orange-red, P–; medulla K+ yellow or orange-yellow, P– or P+ pale yellow (near the algal layer), C–, KC–; containing norstictic and connorstictic acids.

###### Habitat and distribution.

Saxicolous, occasionally growing on the coniferous stump. This species is distributed in Qinghai, Sichuan, and Xizang Prov., China.

###### Notes.

*Lobothallia hedinii* is morphologically similar to *L. polypycnidiata* in general thallus appearance. However, the former differs in its gray to brownish red thallus, parallel lobes of consistent width, and its relatively sparse pycnidia. Chemically, although both species contain norstictic and connorstictic acids, they are distinguished by their medullary spot-test reactions: *L. polypycnidiata* exhibits P– or P+ pale yellow (only near the algal layer), whereas *L. hedinii* shows a distinct P+ yellow reaction. Furthermore, the species differ in reproductive development: *L. polypycnidiata* specimens consistently show scarce ascus maturation despite the apothecial density, while producing abundant pycnidia; in contrast, *L. hedinii* displays significantly higher ascus production but fewer pycnidia. Additionally, *Lobothallia praeradiosa* could be confused with *L. polypycnidiata*, but differs in its smoother upper surface, loosely adnate lobes with partially overlapping margins, orange-brown apothecial margin, and punctiform pycnidia (Kou et al. 2013; Paukov et al. 2019).

###### Additional specimens examined.

China • Qinghai Prov.: Yushu Tibetan Autonomous Prefecture, Qumalai Co., Qumalai Vil., 34°54'53.01"N, 94°46'17.29"E, alt. 4412 m, on rock, 17 September 2020, Xin-Yu Wang et al. XY20-753 (KUN-L 78517), Li-Song Wang et al. 20-68344 (KUN-L 76525), 20-67160 (KUN-L 75336) • Zhiduo Co., Zhiqu Vil., 34°00'44.49"N, 95°47'45.85"E, alt. 4171 m, on rock, 18 September 2020, Li-Song Wang et al. 20-68419 (KUN-L 76601), 20-68413 (KUN-L 76595), Xin-Yu Wang et al. XY20-808 (KUN-L 78572) • Zaduo Co., Angsai Vil., 32°50'52.90"N, 95°28'23.05"E, alt. 4051 m, on rock, 17 September 2020, Li-Song Wang et al. 20-67303 (KUN-L 75480), 20-67265 (KUN-L 75442) • Zhaqing Vil., 32°57'03.90"N, 95°05'29.65"E, alt. 4166 m, on rock, 18 September 2020, Li-Song Wang et al. 20-68582 (KUN-L 76764), 20-68583 (KUN-L 76765) • Ado Vil., 32°50'52.95"N, 95°28'24.98"E, alt. 4029 m, on dead coniferous wood, 20 September 2020, Li-Song Wang et al. 20-68672 (KUN-L 76855) • Haixi Mongol and Tibetan Autonomous Prefecture, Golmud Ci., 35°04'24.13"N, 94°07'46.04"E, alt. 4419 m, on rock, 17 September 2020, Li-Song Wang et al. 20-67134 (KUN-L 75310) • Sichuan Prov.: Garze Tibetan Autonomous Prefecture, Shiqu Co., Sexu Town, 33°07'53.16"N, 97°41'46.91"E, alt. 4214, on rock, 21 September 2020, Li-Song Wang et al. 20-68728 (KUN-L 76911) • Xizang Prov.: Langkazi Co., entrance to Karola Glacier, 28°53'54.24"N, 90°13'31.31"E, alt. 4758, on rock, 24 July 2019, Xin-Yu Wang et al. XY19-2271 (KUN-L 73043) • Chamdo Ci., Jiangda Co., Tongpu Vil., 31°38'30.46"N, 98°26'11.62"E, alt. 3948 m, on limestone, 23 September 2020, Li-Song Wang et al. 20-68878 (KUN-L 77062), 20-68840 (KUN-L 77024).

##### ﻿ Lobothallia pseudopruinosa

Taxon classificationFungiPertusarialesMegasporaceae

Lun Wang & Y. Y. Zhang sp. nov.

2FC09533-E0BA-5678-BA13-9D1DB6041FDD

Fungal Names: FN 572945

Fig. 5

###### Etymology.

The epithet indicates that this species is similar to *Lobothallia pruinosa* in morphology.

###### Diagnosis.

This species is characterized by its tightly adnate and thin thallus, areolate center and shortly lobate margin; plane, white or brownish gray upper surface with fine granular pruina; cryptolecanorine apothecia; large ascospores (10.0-)11.8–13.3–14.8(-17.0) × (8.0-)8.5–9.3–10.0(-11.0) µm; and its long conidia (5–)6–8(–9) × 1 µm.

###### Holotype.

China • Xizang Autonomous Region: Shigatse Ci., Dingri Co., Zhaguo Vil., 28°35'23.26"N, 86°53'55.21"E, alt. 4320 m, on rock, 27 July 2019, Li-Song Wang et al. 19-64075 (KUN-L 68571).

###### Description.

Thallus tightly adnate to the substrate, up to 3 cm across, centrally areolate, 1–2 mm thick, marginally lobate, 0.3–0.8 mm thick. Areoles (0.3–)0.6–1(–1.5) mm wide, angular to rounded, flat, not constricted at base; interspaces between areoles 0.05–0.1 mm wide. Lobes radiate, short, plane, apices equal to slightly wider than base, 1–2 mm long, base 0.5–1 mm wide, apex 0.5–1.5 mm wide. Upper surface white or pale brownish gray, pruinose, brownish where pruina thin. Upper cortex paraplectenchymatous, 30–50 µm thick, containing dark brown granules (soluble in K); epinecral layer with dense grayish-black granules (insoluble in K), 10–40(–50) µm thick. Algal layer 70–100(–150) µm thick, discontinuous; photobiont chlorococcoid, (6–)8–20 µm diameter. Medulla 0.1–0.5 mm thick, opaque, filled with grayish-black granules (insoluble or partially soluble in K). Lower cortex absent.

Apothecia cryptolecanorine, common, grouped in the center, 1–3 per areole, angular, (0.1–)0.3–0.8(–1.0) mm in diameter; disc concave, brownish to black, matt, pruinose; apothecial margin indistinct, concolorous with the thallus. Exciple narrow, widening to 10–30(–50) μm in the uppermost part. Epithecium, hymenium and subhymenial layers combined (100–)125–150(–175) µm high; epithecium 5–15 µm thick, gelatinous; epihymenium 15–25 µm high, with dark brown to brownish granules (soluble in K), N–; hymenium (70–)90–100(–110) µm high, hyaline, I+ blue; subhymenial layers 50–75 µm high, hyaline, I+ blue; algal layer relatively continuous below hypothecium, 50–70 μm high; paraphyses submoniliform, simple, septate, with 1–3 uppermost cells shorter and wider than the basal cells, 4–6 μm wide (basal cells ca. 2 μm wide); asci clavate, *Aspicilia*-type, hyaline, 8-spored, 60–75 × 15–25 µm; ascospores simple, hyaline, broadly ellipsoid, rarely spherical, (10.0-)11.8–13.3–14.8(-17.0) × (8.0-)8.5–9.3–10.0(-11.0) µm (n = 86), l/w ratio (1.0-)1.2–1.4–1.6(-2.1), wall ca.1 µm thick. Pycnidia rare, punctiform, slightly convex, 0.05–0.1 mm diameter, ostiole brown to dark brown, conidia bacilliform, hyaline, (5–)6–8(–9) × 1 µm.

**Figure 5.**
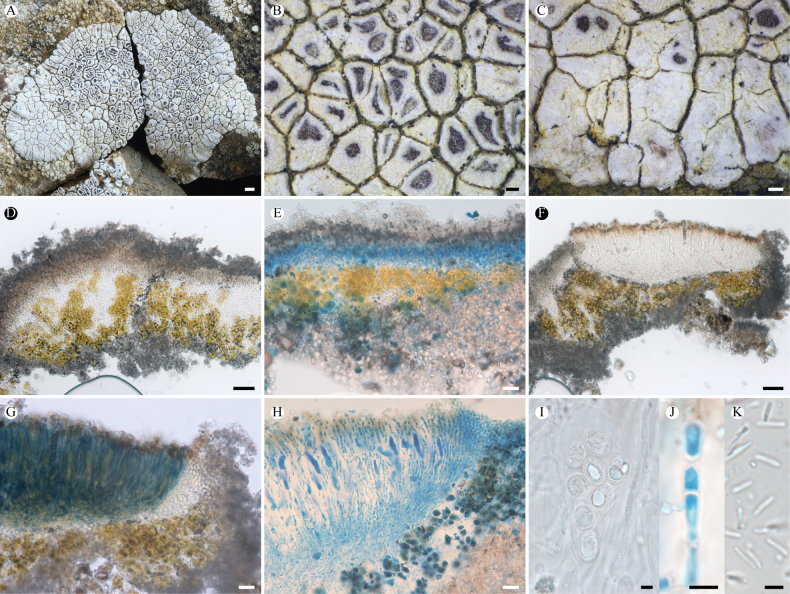
*Lobothallia pseudopruinosa* (KUN-L 68571, holotype). **A.** Thallus; **B.** Areoles and apothecia; **C.** Lobes; **D.** Section of thallus; **E.** Section of thallus (LCB); **F.** Vertical section of apothecium; **G.** Vertical section of apothecium (Lugol’s solution); **H.** Vertical section of apothecium (LCB); **I.** Ascus and ascospores; **J.** Paraphyses (LCB); **K.** Conidia. Scale bars:1 mm (**A**); 0.2 mm (**B, C**); 50 µm (**D, F**); 20 µm (**E, G, H**); 5 µm (**I, J, K**).

###### Chemistry.

Cortex K+ pale yellow or orange, P–. Medulla K+ yellow to orange-red, P+ yellow, C–, KC–. Containing norstictic and connorstictic acids.

###### Habitat and distribution.

Saxicolous. Currently known only in Qinghai, Xizang Province, China.

###### Notes.

Specimens of this new species were collected from Qinghai and Xizang Provinces, China, at elevations ranging from 3000 to 4500 m. The overall morphology of these specimens is uniform, with the exception of voucher 20-67831 (KUN-L 76010), which exhibits a thinner upper cortex composed of loosely arranged hyphae.

Phylogenetically, *Lobothallia pruinosa* and *L. densipruinosa* are the closest relatives of the new species. *L. pruinosa* can be distinguished by its lecanorine apothecia at maturity and broader lobes. *L. densipruinosa* differs in its whitish to light greenish gray and entirely pruinose thallus, apothecial disc dark olivaceous when wet and blackish when dry, and the absence of connorstictic acid (Kou et al. 2013; Ashraf et al. 2022). *Lobothallia pakistanica* resembles *L. pseudopruinosa* in its white, lobate thallus but differs in possessing larger central areoles, broader marginal lobes, and lacking of secondary metabolites (Zulfiqar et al. 2022).

Within *Lobothallia*, *L. brachyloba*, *L. platycarpaL. pulvinata* and this new species share an areolate thallus with lobate margins, a whitish to whitish-gray upper surface, and the presence of norstictic acid. Nevertheless, each can be distinguished by specific characters: *L. brachyloba* has sparse thalline and 1–7 apothecia per areole (Paukov et al. 2019); *L. platycarpa* possesses larger areoles, fewer and non-aggregated apothecia, and limited distribution in North Africa (Algeria) (Zulfiqar et al. 2022); And *L. pulvinata* exhibits a thinner thallus and upper cortex (10–15 µm), and lecanorine apothecia (Zulfiqar et al. 2023). Additionally, *Lobothallia cheresina*, *L. controversa*, and *L. lacteola* also have a tightly adnate, whitish to whitish–gray thallus, but these species differ from *L. pseudopruinosa* in their absent or indistinct marginal lobes and their different secondary metabolites.

###### Additional specimens examined.

*Lobothallia pseudopruinosa*. China • Xizang: Shigatse Ci., Dingri Co., Zhaguo Vil., G219, 28°35'09.99"N, 87°03'42.35"E, alt. 4306 m, on surface of weathered shale in open area, 16 June 2022, Li-Song Wang et al. 22-71222 (KUN-L 85846), Yan-Yun Zhang ZYY22-324 (KUN-L 81905), ZYY22-326 (KUN-L 81907) • Angren Co., 29°19'01.32"N, 87°01'59.60"E, alt. 4524 m, on rock, 19 July 2019, Li-Song Wang et al. 19-63621 (KUN-L 68115) • Qinghai Prov.: Yushu Tibetan Autonomous Prefecture, Qumalai Co., Qumahe Vil., 34°54'50.67"N, 94°46'20.74"E, alt. 4396 m, on limestone, 17 September 2020, Li-Song Wang et al. 20-68331 (KUN-L 76512) • Zeku Co., Maixiu Town, 35°15'53.57"N, 101°52'30.23"E, alt. 3143–3163 m, on rock, 8 July 2022, Xin-Yu Wang and Min Ai XY22-1089 (KUN-L 84907), XY22-1088 (KUN-L 84906), An-Cheng Yin and Han-Xiang Chen 22-72438 (KUN-L 87064) • Jiuzhi Co., NianBaoYuZe National Geopark, 33°13'57.57"N, 100°55'58.56"E, alt. 4063 m, on sandy soil, 8 September 2020, Li-Song Wang et al. 20-67831 (KUN-L 76010).

*Lobothallia pruinosa*. China • Nei Mongol: Bayanhot Town, Helan Mountain, alt. 1600 m, on rock, 17 August 2011, H.Y.Wang 20123575, 20123154, 20123282, 20123626 (SDNU) • Urat Rear Banner Co., alt. 1600 m, on rock, 19 August 2011, H.Y.Wang 20122917 (SDNU), D.B.Tong 20123276, 20123888 (SDNU) • Qinghai Prov.: Hualong Hui Ehtnic Autonomous Co., Yashiga Town, 36°03'52.78"N, 101°57'03.44"E, alt. 2002 m, on rock, 8 July 2022, Li-Song Wang et al. 22-73170 (KUN-L 87648).

##### ﻿ Lobothallia pulchra

Taxon classificationFungiPertusarialesMegasporaceae

Lun Wang & Y. Y. Zhang sp. nov.

A4CEEAB7-4924-556D-8D9A-21E44BB224E1

Fungal Names: FN 572946

Fig. 6

###### Etymology.

The epithet refers to the beautiful morphology of the thallus of this species.

###### Diagnosis.

Thallus areolate with radiate lobes, upper surface white and pruinose; apothecia lecanorine, adnate and rounded, 1–2 apothecia per areole, apothecial margin distinct and persistent, disc plane to slightly convex, not to rarely with faint pruina, containing norstictic and stictic acids.

###### Holotype.

China • Xizang Autonomous Region: Shigatse Ci., Dingri Co., Zhaguo Vil., 28°35'24.49"N, 86°53'58.22"E, elev. 4293 m, on rock, 27 July 2019, Li-Song Wang et al. 19-66027 (KUN-L 70435).

###### Description.

Thallus tightly adnate to the substrate, 0.3–2 mm thick, centrally areolate, marginally lobate. Areoles 0.5–1.2 mm wide, angular to rounded, not constricted at base, interspaces between areoles 0.05–0.1 mm wide. Lobes radiate, short, 1–2.5 mm long, base width (0.3–)0.6–0.8(–1) mm, apex width 0.6–2 mm. Upper surface moderately convex, white, partially pale brown on marginal lobes, pruinose. Upper cortex paraplectenchymatous, even, 30–40 μm thick, containing brown granules (soluble in K); epinecral layer gelatinous, 10–20 μm thick. Algal layer 125–150 μm high, discontinuous; photobiont chlorococcoid, cells 6–18 µm diameter. Medulla 0.3–0.6 mm thick, opaque, filled with gray-black granules (insoluble in K). Lower cortex absent.

Apothecia lecanorine, common, 1–2 per areole, simple to grouped in the center, orbicular to slightly angular by pressure from adjacent apothecia, (0.3–)0.6–2 mm diameter, initially immersed, later sessile, not to slightly constricted at base; disc plane, matt, brownish-black to black, epruinose or faintly pruinose, shallowly fissured when overmature; apothecial margin entire, concolorous with thallus, well-developed and persistent, (0.05–)0.1–0.2(–0.25) mm. Exciple narrow, widening to 70–80 μm in the uppermost part. Epithecium, hymenium and subhymenial layers combined 125–160 µm high; epithecium 5–15 µm high, epihymenium 12.5–20 μm high, filled with dark brown to brown granules (mostly soluble in K), N–; hymenium 90–100 μm high, hyaline, I+ dark blue; subhymenial layers ca. 50 μm high, hyaline, I+ dark blue; discontinuous algal layer beneath hypothecium; paraphyses simple, septate, submoniliform at the tips, 1–4 uppermost cells shorter and broader than basal cells, 3–5 μm wide (basal cells 2–3 μm wide); asci clavate, *Aspicilia*-type, hyaline, 8-spored, 70–80 × 20–30 µm; ascospores simple, hyaline, ellipsoid to broadly ellipsoid, (10.0-)10.8–12.3–13.7(-16.0) × (8.0-)7.8–8.5–9.1(-10.0) µm (n = 30), l/w ratio (1.1-)1.3–1.5–1.7(-1.9), wall ca.1 µm thick. Pycnidia few, punctiform, flat to slightly convex, 0.15–0.2 mm diameter; ostiole brownish black; conidia bacilliform, hyaline, 6–8 × 1 µm.

**Figure 6.**
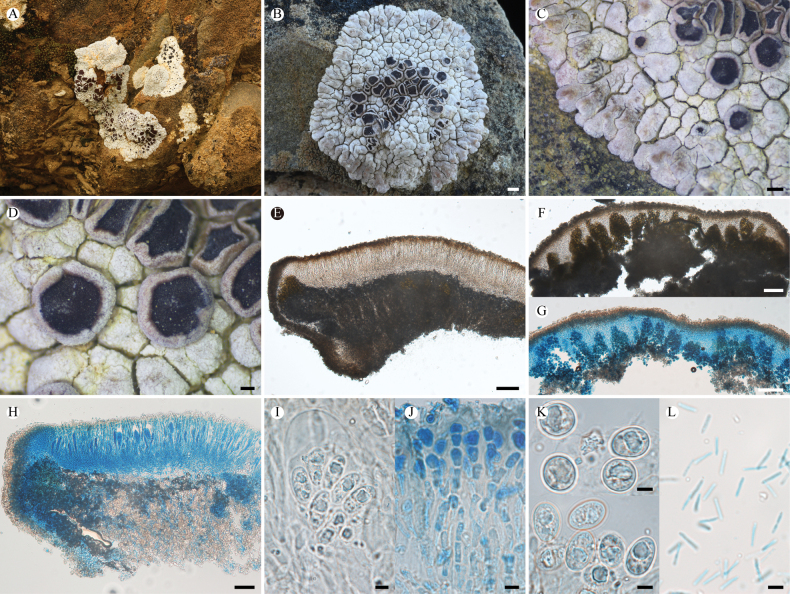
*Lobothallia pulchra* (KUN-L 70435, holotype). **A.** Habitat; **B.** Thallus; **C.** Lobes; **D.** Apothecia; **E.** Vertical section of apothecium; **F, G.** Section of thallus (G in LCB); **H.** Vertical section of apothecium (LCB); **I.** Ascus; **J.** Paraphyses (LCB); **K.** Ascospores; **L.** Conidia (LCB). Scale bars: 1mm (**B**); 0.5 mm (**C**); 0.2 mm (**D**); 100 µm (**E, F, G**); 50 µm (**H**); 5 µm (**I, J, K, L**).

###### Chemistry.

Cortex K+ pale yellow or K–, P–; medulla K+ yellow to orange-red, P+ yellow or orange-red, C–, KC– or KC+ pale yellow; norstictic and stictic acids detected by TLC, some specimens additionally contain cryptostictic, connorstictic and constictic acids.

###### Habitat and distribution.

Saxicolous. Currently known from Xizang and Qinghai Provinces, China.

###### Notes.

*Lobothallia pseudopruinosa* and *L. pruinosa* resemble *L. pulchra* in having an areolate thallus with radiate lobes and a white pruinose surface, but differ in their indistinct apothecial margin, distinctly pruinose discs, and the absence of stictic acid (Kou et al. 2013). *Lobothallia wangii* differs from *L. pulchra* in its predominantly cryptolecanorine apothecia, N+ epihymenium, and the absence of both marginal lobes and stictic acid. *Lobothallia pakistanica* and *L. pulvinata* also have a white thallus with central areoles and marginal lobes, but differ from *L. pulchra* in their thinner thalli, apothecia that are not constricted at base, and in the lack of stictic acid (Zulfiqar et al. 2022, 2023).

###### Additional specimens examined.

China • Xizang Autonomous Region: Tingri Co., Zhaguo Vi., 28°35'24.49"N, 86°53'58.22"E, alt. 4256–4299 m, on rock, 27 July 2019, Li-Song Wang et al. 19-65686 (KUN-L 70194), 19-65652 (KUN-L 70159) • Dagze Co., Bangdui Vi, 29°44'06.51"N, 91°24'55.29"E, alt. 3700 m, on rock, 16 July 2019, Li-Song Wang et al. 19-64597 (KUN-L 69093) • Qinghai: Yushu Tibetan Autonomous Prefecture, Zaduo Co., 32°52'43.64"N, 95°20'29.61"E, alt. 4074 m, on rock, 20 September 2020, Li-Song Wang et al. 20-67281 (KUN-L 75458) • Ado Vi., 32°52'43.35"N, 95°20'30.19"E, alt. 4080 m, on rock, 20 September 2020, Xin-Yu Wang et al. XY20-3169 (KUN-L 79525).

##### ﻿ Lobothallia rubra

Taxon classificationFungiPertusarialesMegasporaceae

Lun Wang & Y. Y. Zhang sp. nov.

6BC5BF55-51D3-56F3-86B3-E10E09BFB4B0

Fungal Names: FN 572947

Fig. 7

###### Etymology.

The specific epithet refers to the red reaction of medulla in C spot-test.

###### Diagnosis.

Thallus areolate with a lobate margin, upper surface white with pale brown tinge, pruinose; areoles raised when bearing apothecia or pycnidia; apothecia lecanorine, disc pruinose; pycnidia protruding; medulla C+ rose–red, contain gyrophoric acid.

###### Holotype.

China • Xinjiang Uygur Autonomous Region: Hejing Co., Baluntai Vil., 42°51'56.45"N, 86°27'09.16"E, alt. 2026 m, on rock, 02 July 2022, Xin-Yu Wang and Min Ai XY22-897 (holotype: KUN-L 84715).

###### Description.

Thallus closely adnate to substrate, up to 5 cm across, areolate, 1–4 mm thick, marginally lobate, 0.4–1 mm thick. Areoles (0.5–)0.8–2(–2.4) mm wide, contiguous, angular to rounded, non-constricted at base, interspaces between areoles 0.1–0.2 mm wide. Lobes slightly broader in the apex, 2–3 mm long, 0.7–1.4 mm wide at base, 0.6–2(–2.5) mm wide at apex. Upper surface convex, cracked, pruinose, white with pale brown tinge where pruina is thin. Upper cortex paraplectenchymatous, 20–40 μm thick, containing brown granules (soluble in K); epinecral layer composed of coarse plate-like crystals, gelatinous material, and gray-black granules (insoluble in K), 10–30 μm thick. Algal layer 75–150 μm high, discontinuous, interrupted by hyphae, 10–60 μm wide; photobiont chlorococcoid, cells 8–20 µm diameter. Medulla 0.5–1 mm high, filled with gray–black granules (generally insoluble in K). Lower cortex absent.

Apothecia lecanorine, common, usually 1 per areole, (0.3–)0.6–1.5(–2.0) mm diameter, rounded, sessile, slightly constricted at base when mature; disc slightly concave to plane, black, pruinose; apothecial margin entire, persistent, 0.1–0.25 mm wide, pruinose, concolorous with thallus. Exciple narrow, up to 40 μm wide. Epithecium, hymenium and subhymenial layers combined (150–)175–225(–250) µm high; epithecium 5–20 µm high; epihymenium 20–50 μm high, filled with dark brown granules (soluble in K), N–; hymenium 100–150 μm high, hyaline, I– or I+ weakly bluish in lower part; subhymenial layers 50–100 μm high, hypothecium 40–50 μm, hyaline, I+ blue; algal layer below hypothecium discontinuous, 50–60 μm high; paraphyses simple, septate, submoniliform at the tips, with 2–3 uppermost cells shorter and broader than basal cells, 3–6 μm wide (basal cells 2–3 μm wide); asci clavate, *Aspicilia*-type, hyaline, 8-spored, 70–85 × 20–30 µm; ascospores simple, hyaline, broadly ellipsoid, (10.0-)11.9–13.1–14.2(-15.0) × (8.0-)8.7–9.4–10.0(-11.0) µm (n = 48), l/w ratio (1.0-)1.3–1.4–1.5(-1.8), wall ca.1 µm thick. Pycnidia common, protruding, 0.1–0.25 mm in diameter, sometimes elongated, 0.5(–0.7) × 0.2 mm (length × width); ostiole brownish to dark brown; conidia bacilliform, hyaline, 5–6 × 1 µm.

**Figure 7.**
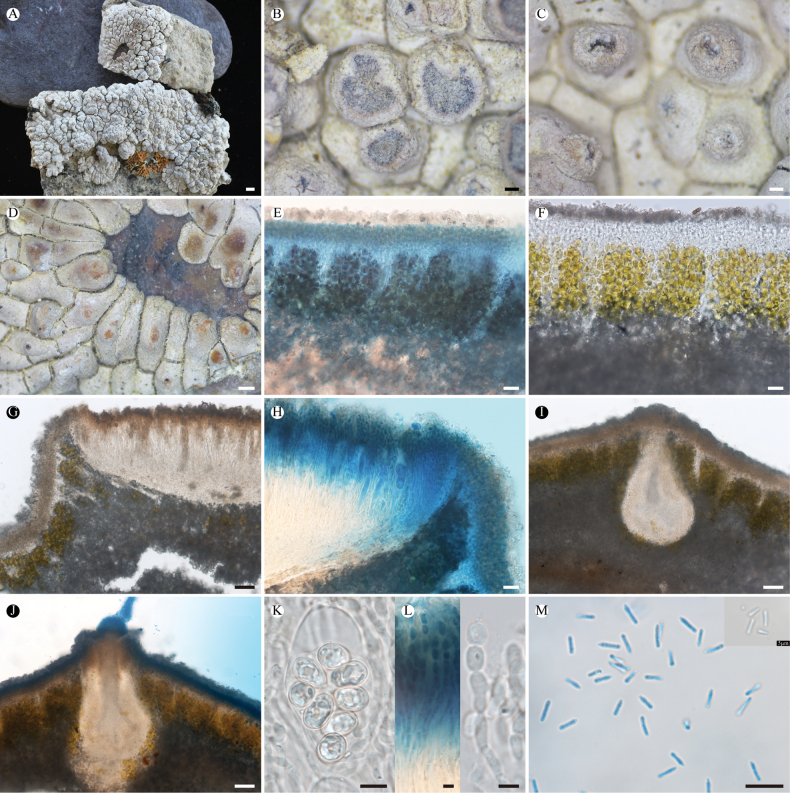
*Lobothallia rubra* (KUN-L 84715, holotype); **A.** Thallus; **B.** Apothecia; **C.** Pycnidia; **D.** Lobes; **E.** Section of thallus (LCB); **F.** Section of thallus (in K); **G.** Vertical section of apothecium; **H.** Vertical section of apothecium (LCB); **I, J.** Vertical section of pycnidium; **K.** Ascus and ascospores; **L.** Paraphyses (left in LCB, right in K); **M.** Conidia (LCB). Scale bars: 2mm (**A**); 0.5 mm (**D**); 0.2 mm (**B, C**); 50 µm (**G**, **I**, **J**); 20 µm (**E, F, H**); 10 µm (**K, M**); 5 µm (**L**).

###### Chemistry.

Cortex: K+ pale yellow, P–; medulla: K+ pale yellow to orange or K–, P–, C+ rose–red, KC+ red to pale yellow; containing gyrophoric, norstictic, stictic, connorstictic and constictic acids.

###### Habitat and distribution.

Saxicolous. Currently known only from Xinjiang, China.

###### Notes.

This species is readily recognized by its C+ rose–red medullary reaction, resulting from the production of gyrophoric acid. *Lobothallia semisterilis* resembles this species in its white to gray thallus and protruding pycnidia, but differs in being terricolous and lacking gyrophoric and stictic acids (Zhang et al. 2020). *Lobothallia pruinosa* shares the characteristic white pruina on both thallus and discs, but differs in its thinner thallus, punctiform pycnidia, and the presence of norstictic and connorstictic acids as the major secondary metabolite (Kou et al. 2013). *Lobothallia pulchra* is similar to *L. rubra* in possessing white thallus and lecanorine apothecia, but differs in having epruinose discs and lacking gyrophoric acid.

###### Additional specimens examined.

China • Xinjiang Uygur Autonomous Region: Hejing Co., Baluntai Town, 42°51'56.45"N, 86°27'09.16"E, alt. 2026–2046 m, on rock, 2 July 2022, An-Cheng Yin and Han-Xiang Chen 22-72301 (KUN-L 86927) • National Highway 216, 42°51'56.39"N, 86°27'09.31"E, alt. 2100 m, on limestone, 2 July 2022, Li-Song Wang et al. 22-72886 (KUN-L 87373).

##### ﻿ Lobothallia stipitata

Taxon classificationFungiPertusarialesMegasporaceae

Lun Wang & Y. Y. Zhang sp. nov.

61101BA9-6EDF-5ACA-A2FB-B6DE73D09338

Fungal Names: FN 572948

Fig. 8

###### Etymology.

The epithet refers to the areoles with a constricted and stipitate base.

###### Diagnosis.

Thallus brown, thick, centrally areolate, areoles with a stipitate base, marginally lobate, margin of lobes free from the substrate with granular pruina on the upper surface; apothecia lecanorine, constricted at the base, disc with conspicuous white pruina, apothecial margin persistent, epruinose, brown to orange-brown; hymenium pale brown.

###### Holotype.

China • Xinjiang Uygur Autonomous Region: Hejing Co., along road G218, 42°54'22.90"N, 86°17'36.95"E, alt. 2228 m, on rock, 01 July 2022, Li-Song Wang et al. 22-72871 (KUN-L 87358).

###### Description.

Thallus relatively loosely attached to the substrate, up to 4–6 cm across, centrally areolate, 3–6(–8) mm thick, marginally lobate, (0.5–)1–2 mm thick, free from the substrate. Areoles angular (0.6–1.2 mm wide) to elongated (1.5–2 × 0.6–1 mm wide), base constricted into a stipe, up to 4 mm long, interspaces between areoles 0.1–0.3 mm wide. Lobes long, simple to 2–3 branched, apices slightly wider than base, 3–5 mm long, 0.6–1.5 mm wide at base, 0.6–2.5 mm wide at apex. Upper surface flat to slightly convex, matt, brown, thinly pruinose, granular pruina present on the margin of lobes. Upper cortex paraplectenchymatous, even, 40–50 μm thick, inspersed with brownish and gray granules (soluble in K); epinecral layer gelatinous, 10–15 μm thick. Algal layer 75–150 μm high, discontinuous; photobiont chlorococcoid, cells 10–25 µm diameter. Medulla 0.6–3 mm high, filled with gray-black granules (generally insoluble in K). Lower cortex absent.

Apothecia lecanorine, common, 1–2 per areole, simple to grouped, orbicular to angular by pressure, (0.3–)0.5–1.5(–1.8) mm wide, constricted at base in maturity; disc concave to plane, brown to black, conspicuously pruinose; apothecial margin entire, persistent, epruinose, brown to orange-brown, 0.1–0.25(–0.3) mm wide. Exciple narrow, widening to 60–80 μm in the uppermost part. Epithecium, hymenium and subhymenial layers combined 125–175 µm high; epithecium 5–15 µm high, with hyaline plate-like crystals; epihymenium 12.5–37.5 μm high, filled with dark brown granules (soluble in K), N–; hymenium 100–120 μm high, pale brown (soluble in K), I+ dark blue; subhymenial layers 50–75 μm high, hypothecium 40–50 μm, pale brown (soluble in K), I+ dark blue; algal layer below hypothecium sparse or absent; paraphyses simple, septate, submoniliform at the tips, with 2–4 uppermost cells shorter and broader than basal cells, 3–5 μm wide (basal cells ca. 2 μm wide); asci clavate, *Aspicilia*-type, hyaline, 8-spored, 70–85 × 18–25 µm; ascospores simple, hyaline, broadly ellipsoid to ellipsoid, (9.0-)9.8–11.4–12.9(-15.0) × (7.0-)6.8–7.6–8.4(-11.0) µm (n = 30), l/w ratio (1.0-)1.3–1.5–1.7(-2.0), wall ca.1 µm thick. Pycnidia punctiform, plane to slightly convex, 0.1–0.25 mm diameter; ostiole brown to dark brown; conidia bacilliform, hyaline, 4–6 × 1 µm.

**Figure 8.**
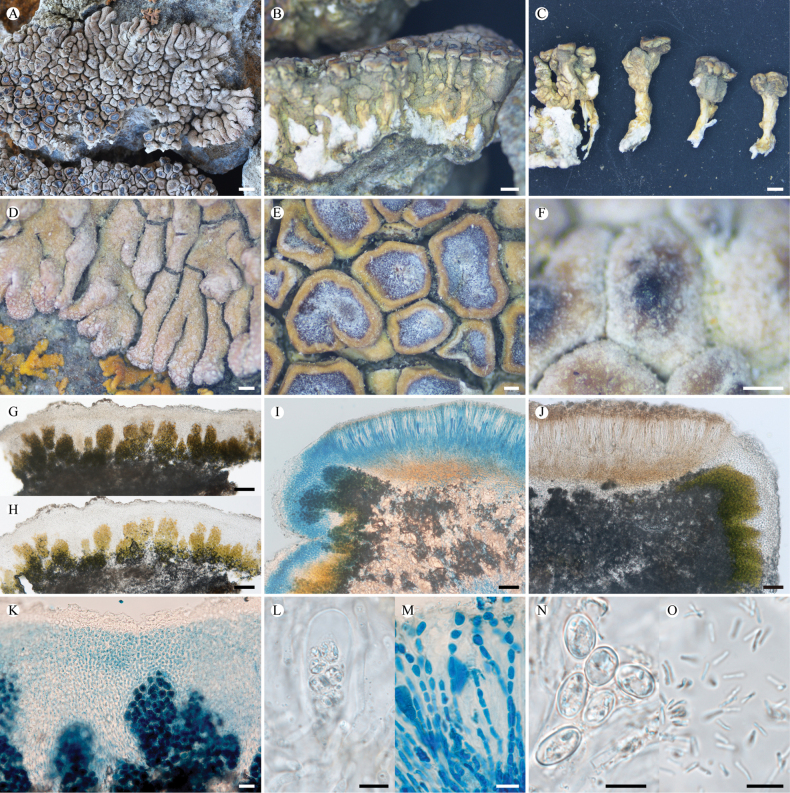
*Lobothallia stipitata* (KUN-L 87358, holotype). **A.** Thallus; **B.** Profile of thallus; **C.** Stipe; **D.** Lobes; **E.** Apothecia; **F.** Pycnidium; **G, H.** Section of thallus (H in K reagent); **I.** Vertical section of apothecium (LCB); **J.** Vertical section of apothecium; **K.** Upper cortex and epinecral layer (LCB); **L.** Ascus; **M.** Paraphyses (LCB); **N.** Ascospores; **O.** Conidia. Scale bars: 2 mm (**A**); 1 mm (**B, C**); 0.5 mm (**D**); 0.2 mm (**E, F**); 100 µm (**G, H**); 50 µm (**I, J**); 20 µm (**K**); 10 µm (**L, M, N, O**).

###### Chemistry.

Cortex K+ pale yellow, P–; medulla K+ yellow, P+ orange, C–, KC–; containing norstictic, stictic, cryptostictic and connorstictic acids.

###### Habitat and distribution.

Saxicolous. Currently only known from Xinjiang Province, China.

###### Notes.

*Lobothallia praeradiosa* resembles *L. stipitata* in having an areolate thallus with radiating lobes, a constricted apothecial base, orange-brown thallus and apothecial margins, and containing norstictic acid. However, the former differs in its epruinose disc, hyaline hymenium, and lack of stictic acid (Kou et al. 2013; Paukov et al. 2019; Ryan 2004). *Lobothallia semisterilis* shares narrowly elongated lobes with granular pruina on the margins, but differs in its terricolous habitat, white to gray thallus, apothecia-like pycnidia, and absence of stictic acid (Zhang et al. 2020).

###### Additional specimens examined.

China • Xinjiang Uygur Autonomous Region: Hejing Co., National Highway 218, 42°54'22.90"N, 86°17'36.95"E, alt. 2228 m, on limestone, 1 July 2022, Yan-Yun Zhang ZYY22-616 (AHUB 00470), ZYY22-617 (KUN-L 82191) • Baluntai Town, 42°54'36.56"N, 86°17'02.47"E, alt. 2171 m, on rock, 1 July 2022, Xin-Yu Wang and Min Ai XY22-887 (KUN-L 84705).

##### ﻿ Lobothallia wangii

Taxon classificationFungiPertusarialesMegasporaceae

Lun Wang & Y. Y. Zhang sp. nov.

0EDCBB2D-92E4-5916-8AEF-C650B0274D75

Fungal Names: FN 572949

Fig. 9

###### Etymology.

The epithet refers to the Chinese lichenologist Li-Song Wang, who collected the type specimen.

###### Diagnosis.

Thallus areolate without marginal lobes, upper surface white, flat; apothecia common, cryptolecanorine to lecanorine, disc black, pruinose, slightly concave to plane, often shallowly fissured at maturity; epihymenium N+ weakly greenish to green, containing norstictic and connorstictic acids.

###### Holotype.

China • Xizang Autonomous Region: Shigatse Ci., Dingri Co., along road G219, 28°35'10.18"N, 87°03'43.62"E, alt. 4311 m, on rock, 06 June 2022, Li-Song Wang et al. 22-71215 (KUN-L 85839).

###### Description.

Thallus tightly adnate to the substrate, 1–4 mm thick, areolate without marginal lobes, spreading in the field up to 25 cm across, the collected specimen is fragmented, up to 4 cm across. Central areoles commonly rounded, (0.2–)0.5–1(–1.6) mm diameter, not constricted at base, interspaces between areoles 0.1–0.2 mm wide; marginal areoles rounded to slightly elongated, 0.8–1.2 × 0.5–1.2 mm. Upper surface flat, white and pruinose. Upper cortex paraplectenchymatous, 30–50 μm thick, inspersed with pale gray granules (soluble in K), uppermost part filled with dark brown granules (partly soluble in K), 20–30 μm thick; epinecral layer gray dark, 20–50 μm thick. Algal layer 75–100 μm thick, mostly continuous, occasionally interrupted by fungal tissue (10–20 μm wide); photobiont chlorococcoid, cells 8–20 µm in diameter. Medulla 0.5–2 mm high, filled with gray-black granules (generally insoluble in K). Lower cortex absent.

Apothecia predominantly immersed and cryptolecanorine, rarely emergent and lecanorine, numerous, 1 to 3 per areole, (0.1–)0.3–1(–1.5) mm wide, simple or grouped, orbicular to angular by pressure; disc black, pruinose, slightly concave to plane, often shallowly fissured at maturity; apothecial margin entire, pruinose, concolorous with thallus, absent when young, appearing and gradually receding as the apothecium develops, (0.05–)0.1–0.2(–0.25) mm wide. Exciple narrow, widening to 40–80 μm in the uppermost part. Epithecium, hymenium and subhymenial layers combined 125–175 µm high; epithecium 5–15 µm high, with hyaline plate-like crystals; epihymenium 15–50 μm high, filled with brown to dark brown granules (dark soluble in K), N+ weakly greenish to green; hymenium (75–)100–125 μm high, hyaline, I+ pale blue to blue; subhymenial layers (40–)50–70(–100) μm high, hypothecium 20–60 μm, hyaline, I+ blue; algal layer below the hypothecium sparse or absent; paraphyses simple, occasionally anastomosing, septate, submoniliform, with 1–3(–4) uppermost cells shorter and broader, 4–6 μm wide (basal cells ca. 2 μm wide); asci clavate, *Aspicilia*-type, hyaline, 8-spored, 60–80 × 20–30 µm; ascospores simple, hyaline, broadly ellipsoid to ellipsoid, (10.0-)11.9–13.4–14.9(-17.0) × (7.0-)7.9–8.6–9.2(-10.0) µm (n = 89), l/w ratio (1.1-)1.4–1.6–1.8(-2.1), wall ca.1 µm thick. Pycnidia few, punctiform, plane to slightly convex, 0.1–0.2 mm diameter; ostiole dark brown; conidia bacilliform, hyaline, 5–7(–9) × 1 µm.

**Figure 9.**
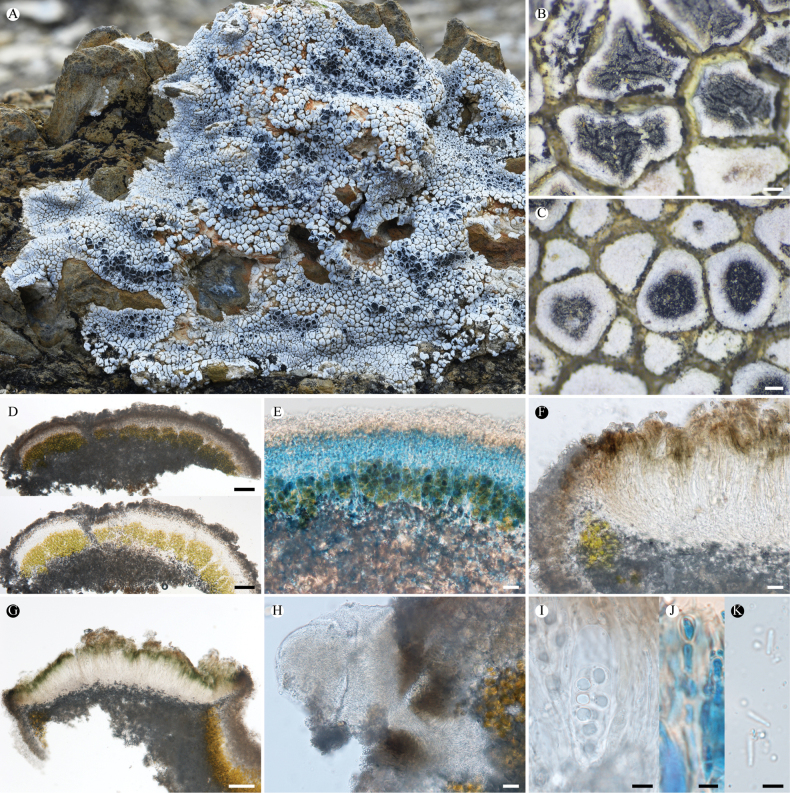
*Lobothallia wangii* (KUN-L 85839, holotype). **A.** Habit; **B, C.** Apothecia; **D.** Section of thallus (below in K reagent); **E.** Upper cortex and epinecral layer (LCB); **F.** Section of apothecium; **G.** Section of apothecium (in N reagent); **H.** Section of pycnidium; **I.** Ascus and ascospores; **J.** Paraphyses (LCB); **K.** Conidia. Scale bars: 1 mm (**A**); 0.2 mm (**B, C**); 100 µm (**D**, **G**); 20 µm (**E, F, H**); 10 µm (**I**); 5 µm (**J, K**).

###### Chemistry.

Cortex K+ pale yellow, P–; medulla K+ yellow to orange-red, P+ yellow, C–, KC–. Norstictic acid and connorstictic acids detected in TLC.

###### Habitat and distribution.

Saxicolous. Currently only known in Xizang, China.

###### Notes.

*Lobothallia controversa* and *L. cheresina* resemble *L. wangii* but differ in having completely immersed apothecia and different secondary metabolites. *Lobothallia controversa* has two chemotypes (Roux et al. 2016): chemotype *controversa* exhibits no reaction to standard reagents (K, C, KC, P, I) but produces terpenoids, while chemotype *reagens* shows K+ and P+ reactions due to the presence of stictic acid complex along with terpenoids. *Lobothallia cheresina* has three chemotypes (Müller 1880; Roux 2012): chemotype *cheresina* lacks secondary metabolites; chemotype *justii* contains stictic acid as the main secondary metabolite; and chemotype *microspora* produces norstictic acid as the major compound. *Lobothallia iqbalii* and *L. lacteola* also possess a white, areolate thallus, and cryptolecanorine to lecanorine apothecia. However, *Lobothallia iqbalii* is distinguished by its plane to convex discs, raised and persistent apothecial margin, N– epihymenium, and absence of secondary metabolites or presence of only norstictic acid (Zulfiqar et al. 2022, 2023). *Lobothallia lacteola* differs from *L. wangii* in its thinner thallus, plicate margins, and absence of connorstictic acid (Paukov et al. 2019).

###### Additional specimens examined.

China • Xizang Autonomous Region: Shigatse Ci., Tingri Co., National Highway 219, 28°35'10.18"N, 87°03'43.62"E, alt. 4311 m, on schist, 16 June 2022, Yan-Yun Zhang ZYY22-324 (KUN-L 81905), ZYY22-331 (KUN-L 81912), ZYY22-334 (KUN-L 81915) • Zhaguo Vi.: 28°35'25.56"N, 86°53'56.03"E, alt. 4292 m, on rock, 27 July 2019, Li-Song Wang et al. 19-65657 (KUN-L 70164), 19-64063 (KUN-L 68559).

#### ﻿Newly recorded species in China

##### ﻿ Lobothallia brachyloba

Taxon classificationFungiPertusarialesMegasporaceae

Paukov & I.V. Frolov, Lichenologist 51(4): 306 (2019).

325ADB75-835E-51F5-B2A8-A19506F8D72D

Fig. 10

###### Description.

Thallus areolate with lobate margin, tightly adnate to the substrate, up to 3 cm across, 0.6–2 mm thick. Areoles (0.5–)0.8–1.5(–2) mm wide, irregular to suborbicular, not constricted at base, interspaces between areoles mostly 0.2–0.4 mm wide. Lobes short, simple to 2–3 branched, 2–3 mm long, 0.6–1.5 mm wide at the base, 1–3 mm wide at the apex, mostly 0.6–0.8 mm thick. Upper surface flat to slightly convex, light gray with brownish tinge, thinly pruinose. Upper cortex paraplectenchymatous, even, 30–40 μm thick, with brown granules (soluble in K); epinecral layer 5–20 µm thick, with brown to black granules (insoluble in K). Algal layer 100–125(–200) µm thick, discontinuous; photobiont chlorococcoid, cells 8–18 µm diameter. Medulla 0.4–1.6(–2) mm high, filled with gray-black granules. Lower cortex absent.

Apothecia cryptolecanorine (rarely elevated as lecanorine, not constricted at base), numerous, 1–8 per areole, orbicular to slightly angular, (0.1–)0.2–0.7(–1) mm diameter; disc slightly concave to flat, black, matt, epruinose to faintly pruinose; apothecial margin usually indistinct (when lecanorine, slightly elevated, 0.05–0.2 mm wide). Exciple narrow, widening to 20–30 μm in the uppermost part. Epithecium, hymenium and subhymenial layers combined 175–225 µm high; epithecium 5–15 µm high; epihymenium 15–40 μm high, with brown dark granules (mostly soluble in K), N−; hymenium 120–140 µm high, hyaline, I+ weakly bluish; subhymenial layers 60–75 μm high, hyaline, I+ blue; algal layer beneath hypothecium discontinuous; paraphyses simple, septate, submoniliform at the tips, with 2–3 uppermost cells shorter and wider than the basal cells, 3–5(–6) μm wide (basal cells ca. 2 μm wide); asci clavate, *Aspicilia*-type, hyaline, 8-spored, 60–70 × 15–22 µm; ascospores simple, hyaline, broadly ellipsoid, (9.0-)9.9–10.8–11.7(-12.0) × (7.0-)7.6–8.2–8.9(-10.0) µm (n = 41), l/w ratio (1.1-)1.2–1.3–1.4(-1.6), wall ca.1 µm thick. Pycnidia few, punctiform, plane to slightly convex, 0.05–0.2 mm diameter, occasionally elongate, 0.2–0.4 × 0.05–0.1 mm; ostioles dark brown; conidia bacilliform, hyaline, 5–6 × 1–1.5 µm.

**Figure 10.**
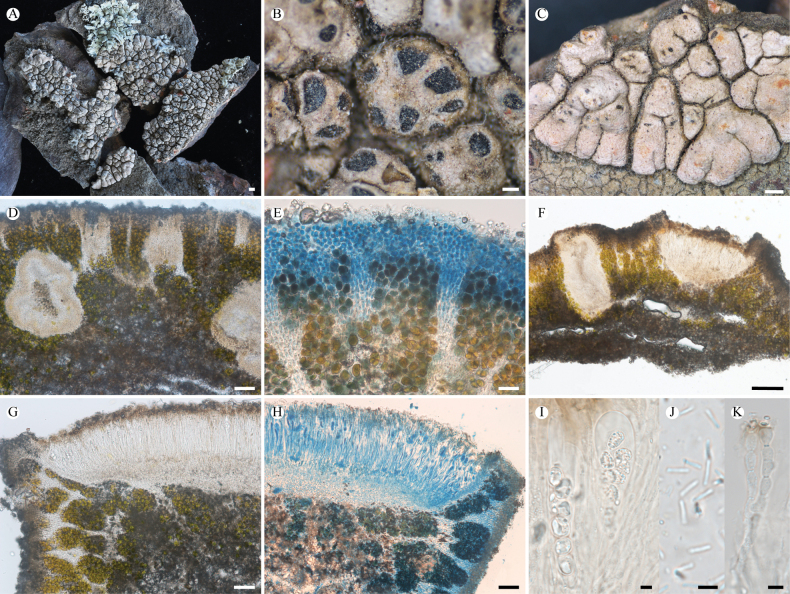
*Lobothallia brachyloba* (22-72815, KUN-L). **A.** Thallus; **B.** Apothecia; **C.** Lobes; **D.** Section of thallus; **E.** Upper cortex and algal layer (LCB); **F.** Section of small apothecium and pycnidium; **G.** Section of apothecium; **H.** Section of apothecium (LCB); **I.** Asci and ascospores (in K); **J.** Conidia; **K.** Paraphyses (in K). Scale bars: 1 mm (**A**); 0.5 mm (**C**); 200 µm (**B**); 100 µm (**F**); 50 µm (**D, G, H**); 20 µm (**E**); 5 µm (**I, J, K**).

###### Chemistry.

Cortex K+ yellow to orange-red, P+ pale yellow; medulla K+ yellow to red, P+ yellow; containing norstictic acid.

###### Habitat and distribution.

Saxicolous. Currently known from the Altai mountains (Republic of Altai, Russia) and the Xinjiang Uygur Autonomous Region (China).

###### Notes.

*Lobothallia brachyloba* is characterized by the areolate thallus with short marginal lobes, the light gray upper surface, the predominantly cryptolecanorine apothecia (1–8 per areole), and the presence of norstictic acid. *Lobothallia crassimarginata* resembles *L. brachyloba* in its gray areolate thallus, short marginal lobes and cryptolecanorine apothecia, but differs in typically bearing only one apothecium per areole, possessing a thick apothecial margin, and containing stictic acid (Kou et al. 2013; Paukov et al. 2019). *Lobothallia benzilanensis* shares the gray thallus and presence of norstictic acid with *L. brachyloba*, but can be distinguished by its dispersed to continuous thallus lacking marginal lobes and its fewer apothecia per areole.

###### Specimens examined.

China • Xinjiang Uygur Autonomous Region: Xinyuan Co., Nalati Town, 43°16'14.26"N, 84°30'08.89"E, alt. 1885 m, on limestone, 1 July 2022, Li-Song Wang et al. 22-72815 (KUN-L 87302).

#### ﻿Type locality species

##### ﻿ Lobothallia hedinii

Taxon classificationFungiPertusarialesMegasporaceae

(H. Magn.) Paukov, Lichenologist 51(4): 312 (2019).

36C20358-11C6-524D-BF9C-48A65657136F

Fig. 11

###### Basyonym.

*Lecanora hedinii* H. Magn. Lichens from Central Asia 1: 98 (1940).

###### Holotype.

China • Gansu Prov.: Eh-ma-ta-ch’üan, 1931, *Bohlin* 55a (S).

###### Description.

Thallus tightly adnate to the substrate, 3–6 cm across (up to 10 cm in intact field specimens), centrally areolate, 3–4 mm thick, marginally lobate, 0.4–1 mm thick. Areoles angular to irregular, not constricted at base, (0.4–)0.6–1(–1.8) mm wide, interspaces between areoles 0.05–0.2 mm wide. Lobes simple, rarely dichotomous, parallel-arranged, (1.5–)2–3(–4) mm long, 0.5–1.5(–2) mm wide. Upper surface plane to slightly convex, gray or pale brown to brownish red, pruinose, pruina usually thicker on the margin of areoles and lobes. Upper cortex paraplectenchymatous, 25–40 μm thick, containing brown granules (soluble in K); epinecral layer 10–15 µm thick, with dark granules (partly soluble in K). Algal layer 125–200 µm thick, discontinuous; photobiont chlorococcoid, cells 10–20 µm diameter. Medulla 0.5–2.5(–3) mm high, filled with gray-black granules (insoluble or partly soluble in K). Lower cortex absent.

Apothecia numerous, initially cryptolecanorine, becoming lecanorine at maturity, 1–3 per areole, adnate, orbicular to slightly angular by pressure, (0.2–)0.4–1.5(–2.2) mm wide; disc matt, brown to black, pruinose, concave to plane; apothecial margin entire, concolorous with thallus, 0.05–0.2 mm wide. Exciple narrow, widening to 50–80 μm in the uppermost part. Epithecium, hymenium and subhymenial layers combined 125–150 µm high; epithecium 10–15 µm high, with plate-like crystals; epihymenium filled with brown granules (soluble in K), N–; hymenium 75–100 µm high, hyaline, I+ blue; subhymenial layers 40–50 μm high, hyaline, I+ blue; algal layer beneath hypothecium discontinuous; paraphyses simple, septate, submoniliform at the tips, with 2–4 uppermost cells shorter and wider than the basal cells, 4–5 μm wide (basal cells ca. 2 μm wide); asci clavate, *Aspicilia*-type, hyaline, 8-spored, 65–75 × 20–30 µm; ascospores simple, hyaline, broadly ellipsoid to ellipsoid, (11.0-)12.5–13.5–14.5(-16.0) × (8.0-)8.4–9.2–9.9(-11.0) µm (n = 33), l/w ratio (1.2-)1.3–1.5–1.7(-1.9), wall ca.1 µm thick. Pycnidia common, punctiform when young, slightly protruding at maturity, 0.1–0.2(–0.5) mm in diameter; ostioles brown to black; conidia bacilliform, hyaline, 5–6 × 1 µm.

**Figure 11.**
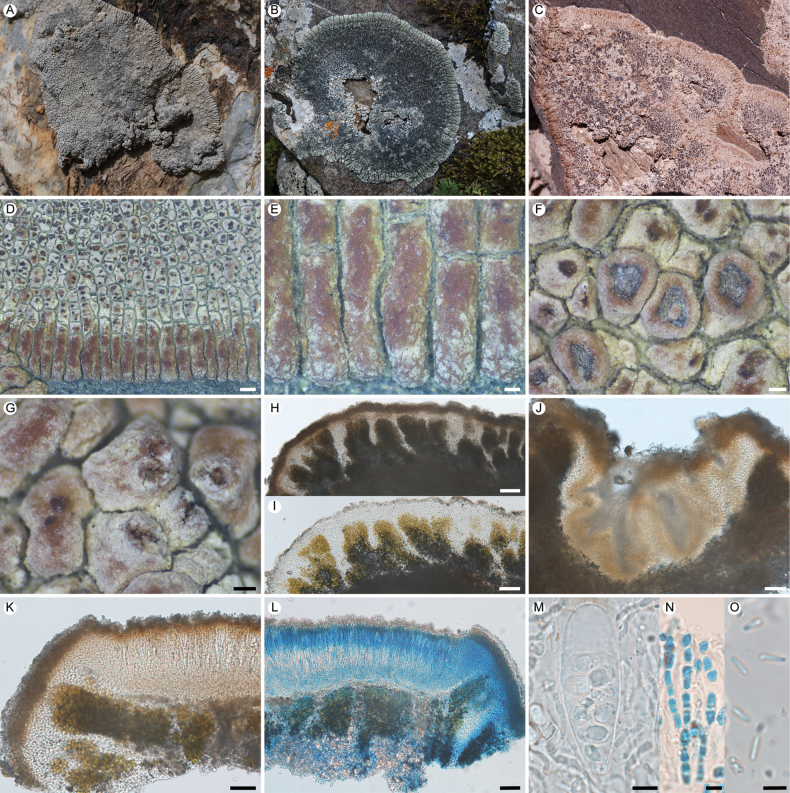
*Lobothallia hedinii***A, B.** Grey upper surface (20-68298, 20-68147); **C.** Pale brown to brownish red upper surface (18-59518). **D–O.** Detail characteristics of 18-59518: **D.** Thallus; **E.** Lobes; **F.** Apothecia; **G.** Pycnidia; **H, I.** Cross-section of thallus (below in K); **J.** Vertical section of pycnidia; **K.** Vertical section of apothecium; **L.** Vertical section of apothecium (LCB); **M.** Ascus and ascospores; **N.** Paraphyses (LCB); **O.** Conidia. Scale bars: 1 mm (**D**); 0.25 mm (**E, F, G**); 100 µm (**H, I**); 50 µm (**J, K, L**); 10 µm (**M**); 5 µm (**N, O**).

###### Chemistry.

Cortex K+ yellow, P–; medulla K+ yellow to orange-red, P+ yellow, C–, KC–; containing norstictic and connorstictic acids, cryptostictic acid also detected in one specimen.

###### Habitat and distribution.

Saxicolous. Currently known from Gansu, Qinghai and Xizang Provinces, China.

###### Notes.

The species was originally described from Gansu Province, China. Our integrated phenotypic and genotypic analyses confirmed its phylogenetic placement for the first time and extended its known distribution to Qinghai and Xizang. Although variable in thallus color, this species can be easily recognized by its areolate thallus with straight and parallel-arranged lobes, and production of norstictic and constictic acids. Its phylogenetic sister species, *Lobothallia polypycnidiata*, resembles *L. hedinii* but differs in having multiple pycnidia per areole, lobes with a slightly broader margin, and the P– or P+ pale yellow (only near the algal layer) medullary spot-reaction, contrasting with the distinct P+ yellow reaction in *L. hedinii*. *Lobothallia praeradiosa* shares production of norstictic and connorstictic acids with *L. hedinii* but differs in its curved and overlapping marginal lobes, gray or orange-brown upper surface, lecanorine apothecia, epruinose discs, and the orange-brown apothecial margins (Magnusson 1940; Kou et al. 2013; Paukov et al. 2019).

###### Additional specimens examined.

China • Gansu Prov.: Yumen City, Yu’erhong Vi., 39°39'29.84"N, 97°10'45.88"E, alt. 3044 m, on rock, 27 May 2018, Li-Song Wang et al. 18-59518 (KUN-L 62904), 18-59517 (KUN-L 62903) • Sunan Yugur Autonomous Co., Dahe Vi., 38°51'29.84"N, 99°38'55.16"E, alt. 2265 m, on sandstone, 6 July 2022, An-Cheng Yin and Han-Xiang Chen 22-72380 (KUN-L 87006) • Qinghai Prov.: Haixi Prefecture, Dulan Co., Xiangjia Vi., 36°00'53.98"N, 97°44'35.61"E, alt. 3036 m, on rock, 15 September 2020, Xin-Yu Wang et al. XY20-2969 (KUN-L 79324) • Dulan Co., S.B. Zhang ZSB24-1727 (KUN-L), Dulan-64 (KUN-L) • Golmud City, 35°53'06.05"N, 94°28'22.55"E, alt. 3665 m, on rock, 16 September 2020, Li-Song Wang et al. 20-68298 (KUN-L 76479), 20-68279 (KUN-L 76460) • Delingha City, S.B. Zhang Dacaidan-9; Hainan Prefecture, Gonghe Co., 36°46'21.59"N, 99°38'47.62"E, 3456 m, Li-Song Wang et al. 18-58220 (KUN-L 61713) • Tianjun Co., 37°38'24"N, 98°37'29"E, alt. 3581 m, on rock, 1 January 2023, S.B.Zhang and H.L.Kang ZSB23-410 (KUN-L 83735) • Yushu Tibetan Autonomous Prefecture, Zaduo Co., 32°52'43.73"N, 95°20'28.82"E, alt. 4083 m, on sandstone, 20 September 2020, Li-Song Wang et al. 20-68620 (KUN-L 76802) • Golog Tibetan Autonomous Prefecture, Gande Co., 33°50'29.51"N, 99°40'33.09"E, alt. 3986 m, on rocks, 13 September 2020, Li-Song Wang et al. 20-68152 (KUN-L 76332), 20-68147 (KUN-L 76327), S.B.Zhang ZSB24-1730 (KUN-L) • Xizang Autonomous Region: Sakya Co., Jiding Town, 29°12'01.18"N, 88°23'10.59"E, alt. 3941 m, on calcareous rocks, 14 June 2022, Li-Song Wang et al. 22-71169 (KUN-L 85793), 22-71155 (KUN-L 85779) • Qushui Co., Qushui Town, 29°23'03.77"N, 90°50'03.23"E, alt. 3580 m, on rocks, 17 July 2019, Li-Song Wang et al. 19-64640 (KUN-L 69137).

### ﻿Key to the species of *Lobothallia* in China

**Table:** 

1	Marginal lobes absent or indistinct	**2**
–	Marginal lobes present and well-developed	**6**
2	Thallus areolate, areoles with smooth upper surface	**3**
–	Thallus rimose-areolate with a rimose upper surface	**5**
3	Apothecia cryptolecanorine or cryptolecanorine to lecanorine, areoles continuous	**4**
–	Apothecia lecanorine, areoles small and dispersed when young, turning larger and continuous at maturity	***L. benzilanensis***
4	Apothecia cryptolecanorine, areoles angular, secondary metabolites absent or stictic/norstictic acids present	***L. cheresina***
–	Apothecia cryptolecanorine to lecanorine, areoles rounded, containing norstictic and connorstictic acids	***L. wangii***
5	Thallus surface off-white. On calcareous rock	***L. determinata***
–	Thallus surface light to dark gray to olive gray. Оn siliceous rock	***L. subdiffracta***
6	Saxicolous	**7**
–	Terricolous, often forming soil crusts with other lichens. Thallus upper surface granular-pruinose, white to gray, lobes narrow and long, apothecia lecanorine, pycnidia punctiform, sometimes protruding as apothecia-like	***L. semisterilis***
7	Secondary metabolites absent	**8**
–	Secondary metabolites present	**9**
8	Lobules present, upper surface pruinose, pruina thicker on the margins of lobes and lobules; disc brown to dark brown, epruinose, shiny; apothecial margin entire	***L. lobulata***
–	Lacking lobules, upper surface white, evenly pruinose; disc black, pruinose, matt; apothecial margin crenulate	***L. crenulata***
9	Medulla C–, gyrophoric acid absent	**10**
–	Medulla C+ rose-red, gyrophoric acid present	***L. rubra***
10	Stictic acid present	**11**
–	Stictic acid absent	**14**
11	Thallus white or gray	**12**
–	Thallus pale brown to brown	**13**
12	Apothecia lecanorine, base constricted at maturity	***L. pulchra***
–	Apothecia cryptolecanorine, not constricted at base	***L. crassimarginata***
13	Lobes short, closely appressed; disc with faint pruina, apothecial margin concolorous with thallus, darkening and receding with age; hymenium hyaline	***L. zogtii***
–	Lobes elongate, loosely attached; disc thickly pruinose, apothecial margin brown to orange-brown, persistent; hymenium pale brown	***L. stipitata***
14	Areoles constricted at base	**15**
–	Areoles not constricted at base	**16**
15	Thallus loosely attached to the substratum, easily detachable, central areoles bullate, lobes strongly convex to almost cylindrical, upper surface whitish-gray to gray, apothecial margin whitish-gray	***L. alphoplaca***
–	Thallus closely adnate to the substratum with only outer of lobes not adherent, areoles and lobes plane to slightly convex, upper surface gray to orange–brown, apothecial margin gray to orangish–brown	***L. praeradiosa***
16	Central thallus thin, less than 2 mm; lobes plane	**17**
–	Central thallus thick, more than 2 mm; lobes slightly convex	**20**
17	Apothecia not exceeding 3 per areole	**18**
–	Apothecia commonly more than 3 per areole	***L. brachyloba***
18	Disc black or dark brown with white pruina	19
–	Disc brown with orange-gray pruina	***L. complanata***
19	Apothecia cryptolecanorine, 0.3–0.8 mm, apothecial margin at the same level with disc	***L. pseudopruinosa***
–	Apothecia cryptolecanorine to lecanorine, 0.7–1.2 mm, apothecial margin somewhat higher than the disc	***L. pruinosa***
20	Pycnidia sparse, punctiform or slightly protruding at maturity	**21**
–	Pycnidia dense per areole, spot-like to elongate and curved	***L. polypycnidiata***
21	Marginal lobes parallel, disc pruinose	***L. hedinii***
–	Marginal lobes non-parallel, disc epruinose	***L. radiosa***

## ﻿Discussion

### ﻿Species diversity

**Figure 12.**
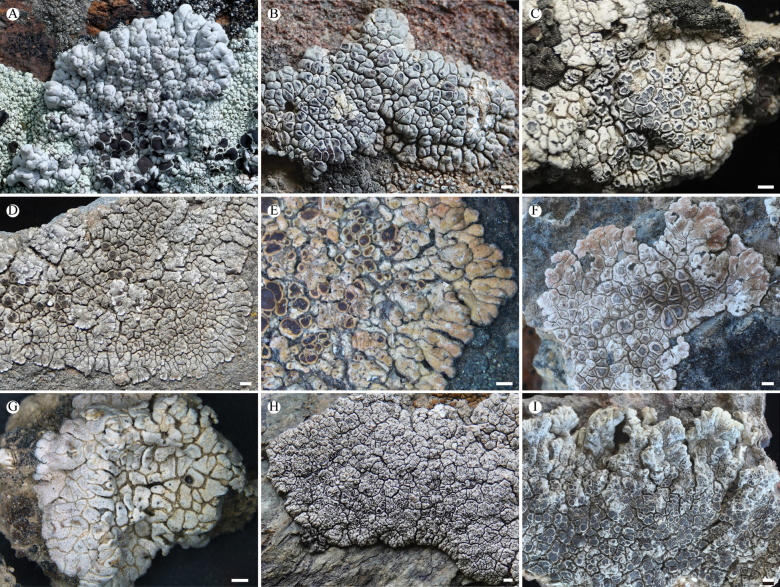
Other *Lobothallia* species from China examined in this study. **A.***L. alphoplaca* (22-72816, KUN-L); **B.***L. crassimarginata* (20122418B, SDNU); **C.***L. crenulata* (ZYY22-301, KUN-L, holotype); **D.***L. lobulata* (ZYY22-819, KUN-L, holotype); **E.***L. praeradiosa* (22-71752, KUN-L); **F.***L. pruinosa* (22-73170, KUN-L); **G.***L. semisterilis* (ZYY22-719, KUN-L); **H.***L. subdiffracta* (ZYY22-628, KUN-L); **I.***L. radiosa* (22-72256, KUN-L). Scales bars: 1 mm (**B–I**).

Thirty-nine species of *Lobothallia* are accepted worldwide in this study. Among them, twenty-two are present in China, including eight new species (Figs 2–9), one newly recorded species (Fig. 10), and thirteen known species, ten of which are studied here (Figs 11, 12) (Magnusson 1940, 1944; Mamut et al. 2010, 2012; Kou et al. 2013; Paukov et al. 2019; Wheeler et al. 2024; Zhang et al. 2020, 2024a). This research significantly increases the species diversity of *Lobothallia* and shows that China is a distribution center of this genus, especially in the north, northwest and southwest. The Qinghai-Tibet Plateau region emerges as a significant diversity hotspot for the genus, exhibiting a substantially higher species richness compared to other areas. Unfortunately, no specimens from northeastern China were examined, but comprehensive future sampling may further enrich the species diversity of *Lobothallia* in the country.

### ﻿Morphological and microscopical characters

In Megasporaceae, *Lobothallia* is characterized by relatively small ascospores that rarely exceed 18 μm in length, and short conidia, 3–10 μm long (Nordin et al. 2010; Kou et al. 2013; Paukov et al. 2019; Zulfiqar et al. 2022). Additional morpho-anatomical characters include: a paraplectenchymatous upper cortex, a discontinuous algal layer; an N- or rarely N+ slightly greenish epihymenium (vs. N+ emerald green in other genera of Megasporaceae) (Paukov et al. 2019); simple to rarely anastomosed paraphyses with swollen, submoniliform to moniliform tips; and a subhypothecial algal layer (Ryan 2004; Kou et al. 2013; Paukov et al. 2019), which may be poorly visible in some cases (Roux et al. 2016).

Although *Antidea brucei* (Owe-Larss. & A. Nordin) T. B. Wheeler, *Aspilidea myrinii* (Fr.) Hafellner and *A. subadunans* (Vain.) T. B. Wheeler, J. W. McCarthy & Fryday possess ascospores and conidia of similar size to the *Lobothallia* species, they are phylogenetically distant (Hafellner and Türk 2001; Owe-Larsson et al. 2007; Wheeler et al. 2024). Similarly, species of *Teuvoa* share anatomical similarities with *Lobothallia*, such as paraphyses structure, ascospore and conidia size. However, *Teuvoa* differs in having non-lobate thalli, lacking secondary metabolites, and its corticolous or terricolous habitat (Rico et al. 2007; Sohrabi et al. 2013b).

The traditional key morphological feature for defining the genus *Lobothallia*—the presence of marginal lobes—exhibits transitional variation across the genus (Hafellner 1991; Ryan 2004). Although it is therefore not a reliable character for generic delimitation, it remains valuable in species-level identification (Paukov et al. 2019). Currently, approximately one-third of the species in *Lobothallia* lack or exhibit indistinct lobes, viz. *L. benzilanensis*, *L. cernohorskyana*, *L. chadefaudiana*, *L. cheresina*, *L. controversa*, *L. determinata*, *L. elobulata*, *L. gangwondoana*, *L. lacteola*, *L. peltastictoides*, *L. recedens*, *L. subdiffracta* and *L. wangii* (Magnusson 1940, 1944; Roux et al. 2016; Paukov et al. 2019; Kondratyuk et al. 2020; Zulfiqar et al. 2022; Wheeler et al. 2024). The length of lobes is a diagnostic character at species level (Paukov et al. 2019; Zulfiqar et al. 2023), ranging from short (< 2 mm) to relatively long (up to 5 mm). Most species of *Lobothallia* have an areolate thallus (at least in the center), with the following exceptions: *L. epiadelpha*, which is centrally squamulose; *L. zogtii*, which is centrally squamulose-areolate (Paukov et al. 2019); and *L. benzilanensis* and *L. gangwondoana*, which contain more or less distant simple areoles (Kondratyuk et al. 2020).

Species delimitation in *Lobothallia* is based on the integration of multiple morphological and chemical characters. Key thallus features for identification include thallus thickness (varying from < 2 mm to 8 mm), areoles size, and the presence of pruina. Apothecial type (e.g., cryptolecanorine, cryptolecanorine to lecanorine, lecanorine), disc color and pruina, and thalline margin width and development are also frequently employed for species delimitation. Additional phenotypic characters further support species delimitation, including habitat conditions and substrate type; thallus attachment, growth form and diameter, the width and depth of inter-areolar cracks; the presence or absence of lobules and of basal constriction in areoles; smooth or rimose upper surface; and the cortex and medulla thickness. Reproductive traits include the number of apothecia and pycnidia per areole; the presence or absence of a constricted apothecial base; disc shape (concave, plane, convex) and texture (shiny/matt, with or without fissures); epihymenium reactions (N− or N+ light green), hymenium and hypothecium colour (hyaline or pale brown), ascospore and conidial sizes (Magnusson 1940, 1944; Ryan 2004; Kou et al. 2013; Nimis 2016; Roux et al. 2016; Paukov et al. 2019; Kondratyuk et al. 2020; Zhang et al. 2020, 2024a; Ashraf et al. 2022; Zulfiqar et al. 2022, 2023).

### ﻿Secondary metabolites

Species of *Lobothallia* can be categorized into two groups based on the presence or absence of secondary metabolites. Among species producing secondary metabolites, norstictic acid is the predominant and most frequently encountered compound (Ryan 2004; Roux 2012; Roux et al. 2016; Kou et al. 2013; Paukov et al. 2019; Kondratyuk et al. 2020; Zhang et al. 2020; Ashraf et al. 2022; Zulfiqar et al. 2022, 2023). Other metabolites include stictic acid (Kou et al. 2013; Moon and Kashiwadani 2009; Roux et al. 2016; Paukov et al. 2019), constictic acid (Ryan 2004; Kou et al. 2013), connorstictic acid (Paukov et al. 2019), salazinic acid (Ryan 2004), and terpenes (Galloway and Ledingham 2012; Roux et al. 2016).

All newly described species in this study contain norstictic acid. Additional secondary metabolites detected include stictic acid in *Lobothallia pulchra*, *L. rubra*, and *L. stipitata*; cryptostictic acid (recorded for the first time in *Lobothallia*) in *L. benzilanensis*, *L. pulchra*, and *L. stipitata*; gyrophoric acid exclusively in *L. rubra*; and connorstictic acid, which frequently occurred as an accessory acid in all new species. Furthermore, thin-layer chromatography (TLC) analysis of 11 known species (10 previously reported and one newly recorded in China) indicated that all species, except *L. crenulata*, *L. lobulata* and *L. subdiffracta*, contain norstictic acid as the major secondary metabolite (Kou et al. 2013; Paukov et al. 2019; Zhang et al. 2024a). Stictic acid was detected in *L. crassimarginata*; connorstictic acid was identified in the species of *L. alphoplaca*, *L. crassimarginata*, *L. hadinii*, *L. praeradiosa*, *L. pruinosa*, and *L. radiosa*.

### ﻿Phylogeny

Although the phylogenetic topology of Megasporaceae from our three-locus dataset is largely congruent with the five-locus results of Wheeler et al. (2024), and both place *Lobothallia* as sister to *Teuvoa*, the statistical support for this relationship is considerably lower in our analysis. Consequently, the relationship between *Lobothallia* and *Teuvoa* remains uncertain and warrants further investigation. Our results confirm that *Lobothallia* comprises three primary clades (Clades I–III), consistent with previous studies (Kou et al. 2013; Paukov et al. 2019; Zulfiqar et al. 2022, 2023; Zhang et al. 2024a). However, the evolutionary relationships among these three clades remain unresolved due to weak supports at the backbone nodes (Fig. 1). The above results, to some extent, support the assumption of Wheeler et al. (2024)—if *Teuvoa* is accepted at the genus level, then a case could be made for erecting additional genera within *Lobothallia*. Whether different clades or subclades of *Lobothallia* warrant recognition as separate genera in the future will depend on comprehensive phylogenetic and morphological studies within the family Megasporaceae.

Furthermore, this study identified six *Lobothallia* species for which molecular data are unavailable: *L. chadefaudiana*, *L. gangwondoana*, *L. kuminovae*, *L. lacteola*, *L. platycarpa*, and *L. zogtii*. Their phylogenetic positions remain unresolved. *Lobothallia chadefaudiana* is morphologically most similar to *L. cernohorskyana*, differing primarily in the absence of secondary metabolites (Clauzade and Vězda 1970; Roux 1977); its phylogenetic position is tentatively inferred to belong to Clade III. *Lobothallia gangwondoana*, characterized by a smooth, slightly brownish-gray or cacao gray thallus with indistinct marginal lobes and distant simple areoles (Kondratyuk et al. 2020), is tentatively placed near *L. crassimarginata* or *L. benzilanensis* within Clade I. *Lobothallia kuminovae*, reported from Siberian Russia, is morphologically more or less identical to *L. praeradiosa* based on its original description (Sedelnikova 1982). Further specimen examination is required to determine whether these two taxa are conspecific. *Lobothallia lacteola*, with its circular outline, plicate margins, and lack of long and straight radial cracks (Paukov et al. 2019), is tentatively inferred to belong to Clade III. *Lobothallia platycarpa* morphologically resembles the subclade comprising the three Pakistan species, *L. pulvinata*, *L. pakistanica* and *L. iqbalii*. *Lobothallia zogtii*, originally described from Xinjiang Province, China, has a brown thallus, short marginal lobes, lecanorine apothecia, and stictic acid (Paukov et al. 2019); its phylogenetic position is tentatively inferred to be within Clade I.

## Supplementary Material

XML Treatment for Lobothallia benzilanensis

XML Treatment for Lobothallia complanata

XML Treatment for Lobothallia polypycnidiata

XML Treatment for Lobothallia pseudopruinosa

XML Treatment for Lobothallia pulchra

XML Treatment for Lobothallia rubra

XML Treatment for Lobothallia stipitata

XML Treatment for Lobothallia wangii

XML Treatment for Lobothallia brachyloba

XML Treatment for Lobothallia hedinii

## ﻿Additional information

### Use of AI

No use of AI was reported.

### Author ORCIDs

Xin-Yu Wang https://orcid.org/0000-0003-2166-6111

Yan-Yun Zhang https://orcid.org/0000-0002-0902-5066

### Data availability

All of the data that support the findings of this study are available in the main text.
